# Humans as inverted bats: A comparative approach to the obstetric conundrum

**DOI:** 10.1002/ajhb.23227

**Published:** 2019-02-27

**Authors:** Nicole D. S. Grunstra, Frank E. Zachos, Anna Nele Herdina, Barbara Fischer, Mihaela Pavličev, Philipp Mitteroecker

**Affiliations:** ^1^ Department of Theoretical Biology University of Vienna Vienna Austria; ^2^ Mammal Collection Natural History Museum Vienna Vienna Austria; ^3^ Department of Integrative Zoology University of Vienna Vienna Austria; ^4^ Division of Anatomy, MIC Medical University of Vienna Vienna Austria; ^5^ Konrad Lorenz Institute for Evolution and Cognition Research Klosterneuburg Austria; ^6^ Cincinnati Children's Hospital Medical Center Cincinnati Ohio; ^7^ Department of Pediatrics University of Cincinnati College of Medicine Cincinnati Ohio; ^8^ Department of Philosophy University of Cincinnati Cincinnati Ohio

## Abstract

**Objectives:**

The narrow human birth canal evolved in response to multiple opposing selective forces on the pelvis. These factors cannot be sufficiently disentangled in humans because of the limited range of relevant variation. Here, we outline a comparative strategy to study the evolution of human childbirth and to test existing hypotheses in primates and other mammals.

**Methods:**

We combined a literature review with comparative analyses of neonatal and female body and brain mass, using three existing datasets. We also present images of bony pelves of a diverse sample of taxa.

**Results:**

Bats, certain non‐human primates, seals, and most ungulates, including whales, have much larger relative neonatal masses than humans, and they all differ in their anatomical adaptations for childbirth. Bats, as a group, are particularly interesting in this context as they give birth to the relatively largest neonates, and their pelvis is highly dimorphic: Whereas males have a fused symphysis, a ligament bridges a large pubic gap in females. The resulting strong demands on the widened and vulnerable pelvic floor likely are relaxed by roosting head‐down.

**Conclusions:**

Parturition has constituted a strong selective force in many non‐human placentals. We illustrated how the demands on pelvic morphology resulting from locomotion, pelvic floor stability, childbirth, and perhaps also erectile function in males have been traded off differently in mammals, depending on their locomotion and environment. Exploiting the power of a comparative approach, we present new hypotheses and research directions for resolving the obstetric conundrum in humans.

## 1 INTRODUCTION

Among mammals, the birth process in humans is strikingly difficult. Human childbirth is long and painful with high rates of morbidity and mortality among mothers and babies, especially if help and modern medical care are not available (Dolea & AbouZhar, 2003; Goldenberg, McClure, Bose, Jobe, & Belizán, 2015; Say et al., 2014). The difficulty arises from the very tight fit between the baby's head and shoulders and the mother's pelvis. Fetal head size and the maternal pelvic dimensions must line up at all points during the birth process, which requires the characteristic rotation of the human fetus (DeSilva & Rosenberg, 2017; Trevathan, 2015). Labor is considered obstructed when the presenting part of the fetus cannot progress into the birth canal, despite strong uterine contractions. The most frequent cause of obstructed labor is cephalopelvic disproportion (CPD, a mismatch between the fetal head and the mother's pelvic brim); other causes include shoulder dystocia, malpresentation or malposition of the fetus (Dolea & AbouZhar, 2003), and altered uterine contractions (Althaus et al., 2006). Rates of CPD are difficult to estimate. In sub‐Saharan Africa, for instance, estimates vary from 1.4% to 8.5% (Dumont, deBernis, Bouvier‐Colle, & Breart, 2001); US statistics suggest rates of 2.3% for infants weighing 3‐4 kg at birth, and 5.8% for those weighing >4 kg (Boulet, Alexander, Salihu, & Pass, 2003). Nevertheless, the global rate of obstructed labor is estimated to be as high as 3‐6% (Dolea & AbouZhar, 2003), despite a system of social and medical care surrounding childbirth in humans.

Birth‐related morbidities are manifold and include immediate injuries due to obstructed labor (eg, fistulas, incontinence) as well as pelvic floor disorders that manifest later (eg, organ prolapse, the descent of organs into or through the vagina). These morbidities can lead to infections and social ostracization (eg, in sub‐Saharan Africa, Arrowsmith, Hamlin, & Wall, 1996; Wall, 1999, 2006). Given the high risk of birth‐related mortality and morbidity to mothers and their babies on the one hand, and the central importance of reproduction to evolution on the other hand, why the human birth canal has evolved to be so narrow is one of the most intriguing evolutionary conundrums today.

### 1.1 Explanations of the obstetric conundrum

Anthropologists and biologists have speculated on the difficulty of human childbirth and pelvic anatomy for many decades, and a number of relevant hypotheses have been formulated. Some are mechanistic in nature and explain the timing of birth, and thus the size of the neonate, as a result of limited maternal metabolic resources (Dunsworth et al., 2012), or they emphasize the effect of recent ecological changes and developmental plasticity in exacerbating an already challenging condition (the “new obstetrical dilemma”; Wells, 2017; Wells, DeSilva, & Stock, 2012). Other hypotheses, on the other hand, tend to focus on pelvic anatomy and argue the latter constitutes an evolutionary compromise between opposing selective forces: selection for a large brain size and large‐bodied neonates on the one hand, and a narrow pelvis—and thus birth canal—on the other. The evolutionary hypotheses differ, however, concerning the source of selection for a narrow pelvis. Some of these hypotheses are mistakenly juxtaposed in the literature as offering competing explanations, but the “obstetric conundrum” is a complex phenomenon that requires all different levels of explanations, from mechanistic (or proximate; asking “how?”) to evolutionary (or ultimate, asking” what for?”), pertaining to both the maternal and the offspring's contribution to the narrow fit of the neonate relative to the birth canal. In this article, we focus on evolutionary hypotheses about pelvic anatomy.

The classic evolutionary explanation for the tight fit between the size of the neonate and the maternal birth canal—the “obstetrical dilemma”—was offered by Washburn (1960). By at least 4 million years ago, bipedality had evolved in the human lineage, long before brain size started to increase about 2 million years ago. The increasingly large‐headed neonates thus had to be delivered through a pelvis that had previously become adapted to bipedalism (Krogman, 1951). Washburn proposed that a wider pelvis would be disadvantageous for bipedal locomotion, hence constituting a selective force opposed to that of obstetrics (see also Lovejoy, 2005a, 2005b; Rosenberg & Trevathan, 1995). To walk upright in an energetically efficient manner with minimal risk of injury, the pelvis must be robust and have a form that maximizes muscle lever arms and minimizes load (Berge, Orban‐Segebarth, & Schmid, 1984; Lovejoy, 2005a, 2005b; Lovejoy, Heiple, & Burstein, 1973; Ruff, 1998; Saunders, Inman, & Eberhart, 1953; Stern & Susman, 1983). Bipedalism‐related changes also affected the position and the shape of the lower half of the pelvis in humans, which dictates the shape of the birth canal (Rosenberg & Trevathan, 1995, 2002).

Despite being considered an attractive explanation for decades, the obstetrical dilemma has only been tested in recent years. Warrener, Lewton, Pontzer and Lieberman (2015) and Vidal‐Cordasco, Mateos, Zorilla‐Revilla, Prado‐Nóvoa, and Rodríguesz (2016) showed experimentally that pelvic size does not predict abductor mechanics or locomotor cost and that women and men are equally efficient at both walking and running, despite women having wider pelves. Wall‐Scheffler (2012) and Wall‐Scheffler and Myers (2017) argued that a wider pelvis allows human females to maintain speed flexibility by decreasing the curvature of the optimal energy walking curve. The kinematic studies by Gruss, Gruss, and Schmitt (2017) and Whitcome, Miller, and Burns (2017) also did not clearly support Washburn's hypothesis. However, definitive conclusions are premature, as these studies could not control for a number of relevant variables, for example, the metabolic profile and fatigue development rate vary considerably across individuals, and indirect calorimetry, an often‐used proxy of energy expenditure, has been shown to be notoriously unreliable (Webb, Annis, Troutman, & Troutman, 1980).

**Figure 1:**
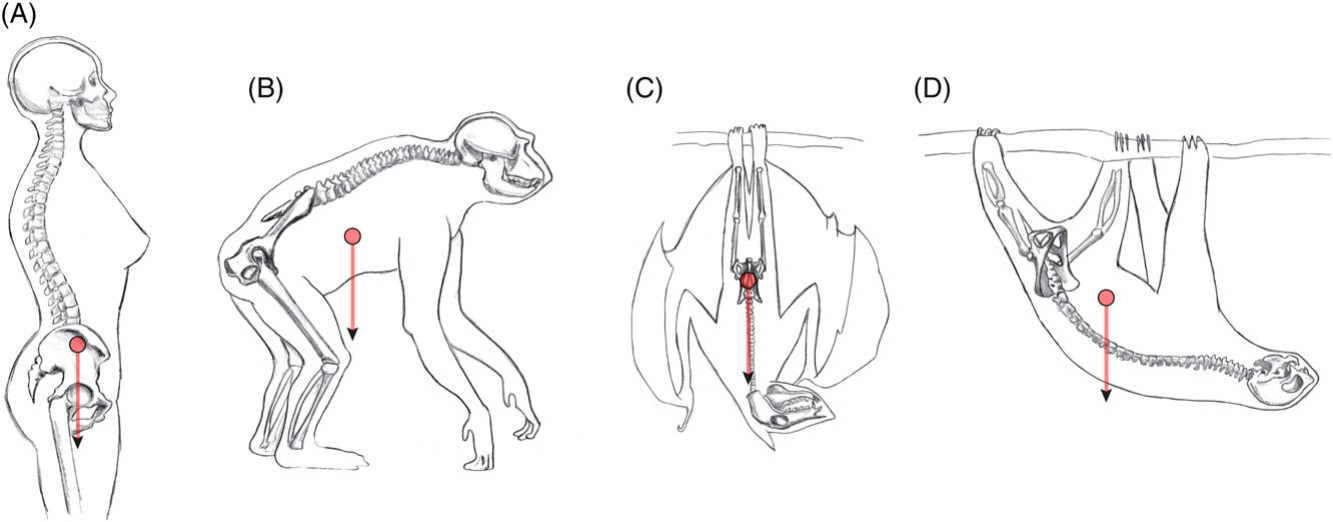
Pelvic orientation, the approximate position of the trunk's center of mass, and the direction of gravity in A, a human; B, a chimpanzee; C, a fruit bat; and D, a sloth, depicted in postures typical for each species. In humans the gravitational force of the visceral organs is exerted directly onto the pelvic floor. In bats and sloths, by contrast, no force is exerted by the visceral organs on the pelvic floor due to the different orientation of the pelvis. (A and B after Whitcome et al., 2017)

An alternative hypothesis, the pelvic floor hypothesis, attributes the selective pressure for a narrow pelvis to the viscera whose weight is supported by the muscles of the pelvic floor in upright animals (Figure 1; Abitbol, 1988; Brown, Handa, Macura, & DeLeon, 2012; Schimpf & Tulikangas, 2005). In primates, the pelvic floor muscles primarily evolved for moving the tail, whereas in upright humans the muscles and fasciae of the pelvic diaphragm create a complex multilevel structure that supports the abdominopelvic organs, which are now vertically aligned with the birth canal (Abitbol, 1988; Elftman, 1932). This support function is enhanced by maintaining a relatively small pelvic outlet, together with the internal bending of the coccyx and the protrusion of ischial spines into the central space of the lower pelvis, thus narrowing the birth canal even further. This hypothesis finds some support from the observation that the ischial spines are most prominent in modern humans, less prominent in fossil hominids, and least prominent (as well as protruding posteriorly, not medially like in humans) in non‐human primates and dogs (Abitbol, 1988). Further corroboration of this hypothesis comes from medical studies showing increased incidence of prolapse and incontinence in women with a wider pelvis (Brown et al., 2012; Handa et al., 2003; Sze, Kohli, Miklos, Roat, & Karram, 1999).

Another line of explanation refers to the adaptation of body form of endothermic animals in response to thermoregulation (Bergmann, 1847; Mayr, 1956; Meiri & Dayan, 2003). Bergmann's rule is applied to explain the elongated body proportions of some African populations: increasing their surface‐to‐volume ratio results in effective “heat dumping”, as opposed to groups from high latitudes with shorter limbs and stockier body form, for example, Inuit and Lapland people as well as Neanderthals (Holliday, 1997; Ruff, 1994; Weaver, 2003; Weaver & Hublin, 2009). Climatic adaptation of body form may also have affected the pelvis as the upper portion (the “false pelvis”, the upper and broader part of the pelvic cavity above the pelvic brim) determines the proportions of the lower trunk (Torres‐Tamayo et al., 2018).

These three hypotheses evince the multiple and partly opposing functions that the human pelvis has to serve: childbirth, locomotion, posture, pelvic floor stability, and thermoregulation. In the face of these functional conflicts—documented by the persistently high rates of both obstructed labor and pelvic floor disorders—pelvic form has evolved as a “compromise solution” to accommodate all these demands. The evolutionary dynamics underlying these conflicts are detailed by the cliff‐edge model (Mitteroecker, Huttegger, Fischer, & Pavličev, 2016; Mitteroecker, Windhager, & Pavličev, 2017). Medical data show a continual decrease of morbidity and mortality (ie, increased evolutionary fitness) with increasing neonatal weight and decreasing maternal pelvic width, but only up to the point when the baby no longer fits through the birth canal; then fitness drops sharply in the absence of medical care. In their mathematical model, Mitteroecker et al. showed that the evolutionary stable state for this highly asymmetrical fitness function, imposed on symmetrically distributed pelvic and neonatal dimensions, always entails a certain rate of cephalopelvic disproportion. In fact, even relatively weak selection toward a narrow birth canal suffices to explain the high rates of cephalopelvic disproportion in modern humans. The actual selective forces and the resulting evolutionary “compromises” are likely to vary among human populations due to variation in climatic, ecological, and sociocultural environments, along with genetic drift and differential migration (Betti, 2014, 2017; Grabowski & Roseman, 2015; Wells, 2015; Wells et al., 2012).

Among primates, with their generally precocial (ie, large and mature) young, humans became secondarily more altricial (more premature and helpless offspring). Because of the large human neonate, it has been proposed that this “secondary altriciality” has evolved to alleviate the obstetrical burden (eg, Montagu, 1961; Portmann, 1941; Rosenberg & Trevathan, 2002; Washburn 1960). Similarly, the softening and widening of the pubic symphysis during pregnancy is often considered a human‐specific adaptation to ease childbirth. Yet we will show here that none of these conditions are specific to humans. Numerous other mammals have considerably larger and more prematurely born offspring. Furthermore, many other mammals, including most primates, have a much more flexible symphysis and sacro‐iliac joint than humans.

### 1.2 A comparative mammalian perspective to human childbirth

Due to the multitude of confounding factors, including climatic, ecological, behavioral, and cultural influences, evolutionary hypotheses like the ones reviewed above cannot be adequately tested in a single species, especially not in our own species. Human variation in posture, the bony pelvis, the pelvic floor, and the size of the neonate is too small—and due mainly to environmental (eg, nutritional) differences—to disentangle the various past evolutionary processes. At the same time, modern humans' ecological and sociocultural environments are highly heterogenous and deviate from the environment that has driven our anatomical evolution during the Plio‐Pleistocene.

A comparative study of non‐human primates and other mammals provides a way to study evolutionary processes, adaptive scenarios, as well as functional and phylogenetic constraints underlying childbirth‐related traits. Although the (relatively) high incidence of obstructed labor is likely a uniquely human phenomenon, not all factors that contribute to obstructed labor are unique to humans. There are several other mammalian taxa (sometimes entire groups of species) that have high maternal investment during gestation and give birth to large neonates relative to maternal size. Like humans, other mammals are subject to selective forces exerted by locomotion, posture, and body size, which constrain their pelvic morphology. For instance, the physical impact of the location of the center of mass and the direction of gravity on pelvic hard and soft tissues depends on posture (horizontal as in quadrupeds, or vertical as in bipeds) and orientation (with the head above the limbs, as in most terrestrial quadrupeds and bipeds, or inverted as in sloths or head‐down roosting bats), as illustrated in Figure 1. Likewise, variation in mode and speed of locomotion is associated with differences in the forces of inertia and the impact on the pelvis (eg, acceleration, frequent changes of direction, or slow and steady locomotion). These physical differences are reinforced by variation in overall body size due to the positive allometry of mass with respect to linear body dimensions (see below).

In this perspectives article, we aim to demonstrate the potential of the comparative method in answering questions about human childbirth by briefly reviewing the relevant mammalian diversity in relative neonatal size, positional behavior, and pelvic morphology in relation to the human condition. We frame hypotheses about how these factors may, individually or together, have influenced the evolution of mammalian pelvic morphology, and present first comparative results. We highlight bats, the only mammals to have evolved powered flight, as an interesting candidate for testing the pelvic floor hypothesis owing to relevant similarities and dissimilarities to humans in reproductive parameters, life history, and pelvic morphology.

We combine our literature review with comparative analyses of neonatal and female body and brain mass. To this end, we connected and extended three existing datasets: Tague's (Tague, 2016) data on neonatal and female body mass for 266 mammalian species (Table S1 in Supporting Information); data on adult brain and body mass for 630 mammal species by Boddy et al. (2012a, 2012b); and data on neonatal brain and body mass along with maternal body mass for 109 species by Capellini, Venditti & Barton (2010a, 2010b). Combined, these data represent monotremes, marsupials, and all four major branches of placental mammals with representatives of most of the higher taxa conventionally classified as “orders”—(1) Afrotheria: Hyracoidea (hyraxes), Sirenia (sea cows), Proboscidea (elephants), Macroscelidea (elephant shrews), and Afrosoricida (tenrecs and golden moles); (2) Xenarthra: Pilosa (anteaters and sloths), and Cingulata (armadillos); (3) Euarchontoglires: Rodentia (rodents), Lagomorpha (hares and rabbits), Scandentia (tree shrews), and Primates; and (4) Laurasiatheria: Eulipotyphla (shrews, hedgehogs, and relatives), Chiroptera (bats), Carnivora (carnivorans, incl. Pinnipedia or seals), Pholidota (pangolins), Cetartiodactyla (even‐toed ungulates, incl. Cetacea or whales), and Perissodactyla (odd‐toed ungulates). The lower weight classes are dominated by bats, shrews and rodents, the highest only contains whales. Some analyses were based on smaller subsets of species that were covered by two or all three of the data sets.

**Figure 2:**
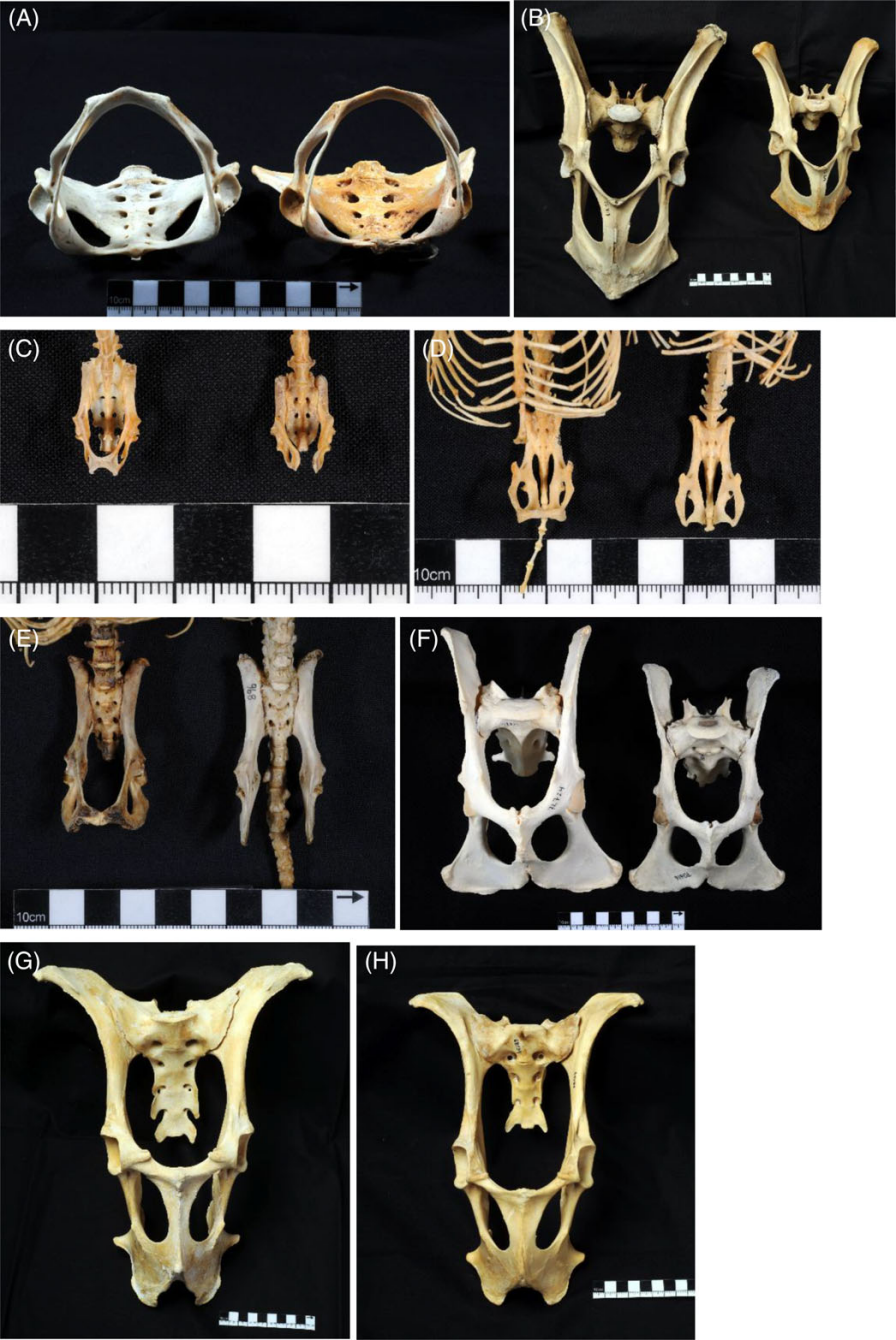
Pelves of selected mammal species. Males on the left, females on the right. Collection numbers are given. A, Choloepus hoffmanni (Hoffmann's two‐toed sloth, Xenarthra: Pilosa), NMW 31566 and NMW 3996. B, Macropus rufus (Red kangaroo, Marsupialia: Diprotodontia), NMW 22717 and NMW 22747. C, Nyctalus noctula (Common noctule, Laurasiatheria: Chiroptera), NMW 42194 and NMW 36120. D, Rousettus aegyptiacus (Egyptian fruit bat, Laurasiatheria: Chiroptera), NMW 20840 and NMW 20834. E, Erinaceus roumanicus (Northern white‐breasted hedgehog, Laurasiatheria: Eulipotyphla), NMW 20153 and NMW 968. F, Acinonyx jubatus (Cheetah, Laurasiatheria: Carnivora), NMW 72724 and NMW 70616. G and H, Rangifer tarandus (Reindeer, Laurasiatheria: Cetartiodactyla), male (G) and female (H), NMW 69121 and NMW 68134. For photographs of pelves of additional mammalian taxa, see Supporting Information

To visualize the diversity in pelvic morphology across mammalian lineages, we took photographs of bony pelves of males and females of various mammalian species representing different clades as well as different morphological adaptations to habitat and locomotion. In particular, we highlight groups with sexual dimorphism in pubic symphysis morphology (Figure 2 and Figures S1‐S22 in the Supporting Information).

## 2 POSITIONAL BEHAVIOR: LOCOMOTION AND POSTURE

Positional behavior describes the postures an animal assumes during or between movements, that is, those associated with locomotion as well as resting (Gebo, 2013). Among extant primates, an upright, obligatorily bipedal mode of locomotion is unique to humans, but a habitual orthograde (upright) posture is not (Fleagle, 2013; Gebo, 2013). Increased pressure on the pelvic floor arising from frequent loading from the viscera during a variety of orthograde positional behaviors may therefore be experienced by several primates and non‐primate mammals. Primate examples include “vertical clingers and leapers” (some small and medium‐sized strepsirrhine lemurs and galagos, tarsiers, and New World monkeys). Among them, indriids are known to move by means of bipedal hopping on the ground (eg, Fleagle, 2013). Furthermore, spider monkeys and gibbons (true gibbons and siamangs) move arboreally by means of brachiation (arm swinging) with the body in a vertical position, and gibbons move bipedally when on the ground (Chivers, 2013; Gebo, 2013). Orangutans are suspensory climbers in an orthograde position (Fleagle, 2013; Gebo, 2013). There are additional monkey taxa that are known to maintain vertical postures while feeding or resting and which may therefore be subject to increased strain on their pelvic floor (eg, sakis, some marmosets, red‐bellied titi monkeys, saddleback tamarins, and gelada baboons). The fact that primates exhibit various combinations of modes of locomotion and postures demonstrates that a primate's posture is not solely determined by the mechanism of progression and, thus, that these factors may have had separate effects on the evolution of pelvic form. Indeed, Lewton and Dingwall (2015) found that, among a taxonomically broad sample of primates, vertical clingers and leapers as well as “non‐slow” suspensory taxa (spider monkeys, gibbons, and orangutans) had, on average, shorter pubic ramus lengths than arboreal and terrestrial quadrupedal primates. Shorter pubic rami reduce the size of the pelvic canal and thus also the area across which the pelvic floor muscles and fasciae stretch; this may be an adaptation to enhance the support function of the pelvic floor.

Obligatory bipedalism is found among non‐primate mammals. Many marsupials, such as kangaroos and wallabies, move by bipedal hopping (preferentially at high speeds), as do placental mammals such as kangaroo rats and mice, hopping and jumping mice, and springhares (Denys, Taylor, & Aplin, 2017; Eldridge & Frankham, 2015; Hafner, 2016; López‐Antoñanzas, 2016; Whitaker Jr, 2017). They may therefore be subject to increased pressure on their pelvic floor, specifically as a result of high‐impact bipedal movements (which may have different effects on the musculoskeletal system than static positions, or even walking and running). Kangaroos, such as the eastern gray kangaroo and the red kangaroo (*Macropus* sp.) are of particular interest because they have an adult body size that overlaps with that of humans (17‐90 kg; Eldridge & Frankham, 2015). Moreover, they exhibit strong sexual dimorphism in overall body size within species, and have close relatives that are smaller‐bodied; the intra‐ and interspecific variation in body size (but not in positional behavior) thus allows the allometric effect of visceral loading onto the pelvic floor to be tested in these bipedal marsupials. Because marsupials give birth to very small neonates relative to maternal size after a short gestation period (Eldridge & Frankham, 2015), the bony pelvis does not pose a constraint to successful parturition. In the case of such virtually absent obstetric selection, the impact of orthograde positional behaviors and their associated gravitational force on the pelvic floor can be studied independently of human‐specific locomotion and the consequences of childbirth. Figure 2B shows the marked sexual dimorphism in pelvic size, especially in pelvic height, in the red kangaroo (*Macropus rufus*), resulting from the dimorphism in overall body size, as well as the comparatively narrow pelvic width in both sexes.

Quadrupedal species may also incur high forces on their musculoskeletal system, including the pelvic floor. Cursorial species, such as many even‐toed (Cetartiodactyla, eg, Bovidae) and odd‐toed ungulates (Perissodactyla, eg, *Equus*), as well as large carnivores (Carnivora, eg, lions and cheetahs) engage in maneuverable, high‐speed running (and sometimes jumping, as in impala and springbok), which results in high‐impact forces on their skeleton and pelvic floor. A change in either speed or direction (ie, velocity) constitutes acceleration, for which forces need to be applied to the body by the muscles. Consequently, other parts of the body, such as the abdominopelvic organs, exert force on their surrounding hard and soft tissue (such as the pelvic floor) as their velocity changes. The fused pubic symphysis of reindeer and cheetahs may constitute such an adaptation to fast, high‐impact locomotion (Figure 2F‐H).

In contrast, there are slow‐moving mammals that assume pronograde (horizontal) positions, which therefore are not subject to high forces associated with acceleration. Among placental mammals, such species notably include sloths (Xenarthra: Pilosa) and lorises (Euarchontoglires: Primates), that is, slow and slender lorises, pottos, and angwantibos. These taxa engage in slow, deliberate arboreal quadrupedalism, which is inverted the majority of the time for sloths and frequently for lorises (ie, the body is below the limbs as during most horizontal below‐branch suspensory behaviors) (eg, Ashton & Oxnard, 1964; Dykyj, 1980; Morraes‐Barros, 2018; Pauli, 2018; Roonwal & Mohnot, 1977 ; Walker, 1969). In both lineages, a ventrad elongation of the pubic rami increases the mechanical advantage of the abdominal musculature required for slow arboreal suspension (Lewton & Dingwall, 2015). However, this pubic elongation also extends the dimensions of the pelvic canal and thus the pelvic floor area. Figures 1 and 2A illustrate this condition in the sloth: the pubic rami are long and fully fused to form a bony ring that projects far ventrally, and consequently the pelvic canal is very large. The absence of sexual dimorphism in pelvic in‐/outlet size in sloths (Figure 2A and Figure S20)—along with relatively small neonatal masses (Tague, 2016)—suggests that the observed pubic morphology is a locomotory adaptation to their particular ecological strategy; not an adaptation to childbirth. Due to below‐branch suspension, the sloth's pelvis is oriented in a horizontal to near‐vertical, “upside down” position, with gravity acting away from the pelvic floor (Figure 1D). Combined with their slow and deliberate form of arboreal quadrupedalism, they likely incur very low and infrequent pressure on their pelvic floor from the viscera. A similar lack of frequent loading and high‐impact forces on the pelvic floor applies to the slow‐moving and “hanging” (Roonwal & Mohnot, 1977; Walker, 1969) lorises.

We propose that these species are able to “afford” such a large pelvic canal and a presumably vulnerable pelvic floor because of their specific positional behavior that minimizes load on the pelvic floor. Unlike in humans, selection for a large pelvic canal has not been counteracted by selection for pelvic floor stability (Curtis, 1995; Lewton & Dingwall, 2015).

## 3 BODY SIZE IN PLACENTAL MAMMALS

**Figure 3:**
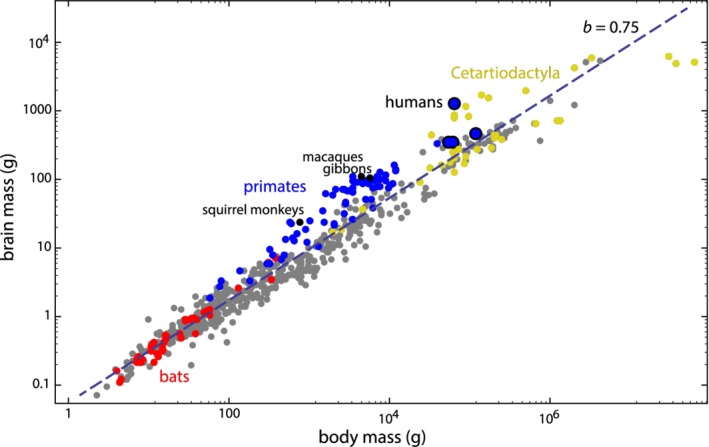
Average brain mass versus body mass for 630 mammalian species (data from Boddy et al., 2012a, 2012b). On this log–log scale, the linear regression has a slope of 0.75, reflecting the well‐known negative allometry of brain mass. Cetartiodactyla: even‐toed ungulates, including Cetacea (whales and dolphins), which are nested within the terrestrial artiodactyls. For the number of species by higher‐order taxon, see Table S2 in Supporting Information

Average body mass varies by eight orders of magnitude in mammals, ranging from a few grams in shrews and small bats up to 100 tons and more in whales. The variation in average brain mass spans five orders of magnitude (from 0.07 g in shrews to 6 kg or more in whales; Boddy et al., 2012b). As is well known, brain mass is negatively allometric (larger mammals tend to have smaller *relative* brain size), with humans being the species with the largest adult brain for their body size (Figure 3). The great apes have the largest brains among non‐human primates in absolute terms, but not in relation to their body mass. In fact, gibbons, macaques, and squirrel monkeys, all of which are known to have occasional obstructed labor (Abee, 1989; Aksel & Abee, 1983; Bowden, Winter, & Ploog, 1967; Debyser, 1995; Stockinger et al., 2011), are among those mammals with the largest *relative* brain size (Figure 3).

These gross differences in body mass not only affect biomechanical demands on the skeleton, postural behavior, and locomotion; they also affect the pressure on the pelvic floor. The weight of the inner organs scales with the third power of body size, whereas pelvic floor area scales with the second power. The vertical pressure (force per unit area) on the pelvic floor thus increases with body size. Similarly, the insertion area of the pelvic floor muscles and ligaments increases only with the second power of body size. For species with a certain degree of habitual orthogrady, one would thus expect that—as an allometric response—larger animals evolved stronger pelvic floor muscles and/or a pelvic form that supports the pelvic floor: that is, a relatively narrower pelvic outlet—and thus a smaller pelvic floor area—resulting in a higher stiffness of the pelvic floor tissue and thus a decrease in the deformation of the pelvic floor under the weight of the inner organs compared to smaller‐bodied species.

Such an allometric relationship has also been found in modern humans. Fischer and Mitteroecker (2015, 2017) showed that taller women tend to have a more oval‐shaped (laterally compressed) pelvic canal and a sacral bone protruding into the birth canal. Shorter women, by contrast, tend to have a rounder (gynecoid) pelvis, which is known to be beneficial for childbirth, and a posteriorly projecting sacrum. On average, shorter women have more difficult births than taller women (Camilleri, 1981; Sheiner, Levy, Katz, & Mazor, 2005); hence, Fischer and Mitteroecker (2015) interpreted this allometric pattern as an adaptive integration of pelvic shape and stature that alleviates the obstetrical burden. But this line of argument can be complemented by an allometric explanation in terms of the scaling of the weight of the visceral organs with body mass, especially because the same association between pelvic shape and stature was also found in males (Fischer & Mitteroecker, 2015, 2017). Compared with shorter individuals, taller individuals experience a disproportionately higher pressure on the pelvic floor and thus would more greatly benefit from a relatively narrow pelvis and an inwardly protruding sacrum.

Again, such an evolutionary hypothesis cannot be easily tested in modern humans alone. Future research may utilize comparisons of pelvic form in related taxa with very different body sizes, for example, kangaroos and wallabies, kangaroo rats, and springhares.

## 4 NEONATAL BODY SIZE

**Figure 4:**
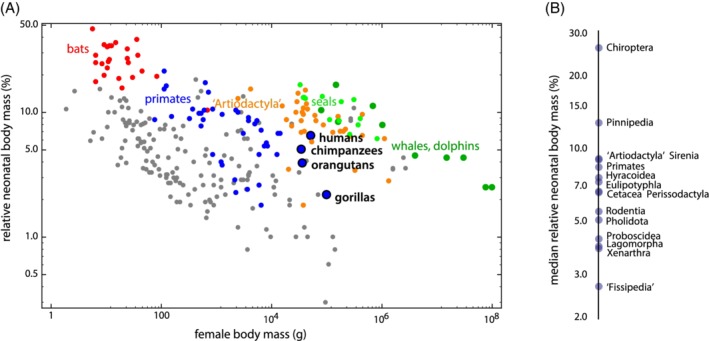
A, Relative neonatal body mass (neonatal mass as percentage of maternal mass) versus maternal body mass for 284 mammalian species on a log‐log scale (data from Tague, 2016 and other literature sources, see Supporting Information Table S1). B, Ranking of different mammalian groups according to their relative neonatal body mass. “Fissipedia” refers to the paraphyletic group of terrestrial carnivorans, that is, Carnivora excl. Pinnipedia (seals). Similarly, “Artiodactyla” refers to the paraphyletic terrestrial even‐toed ungulates, that is, Cetartiodactyla excl. Cetacea (whales and dolphins). For the number of species by higher‐order taxon, see Table S2

Not only absolute body mass, also the body mass of the neonate relative to that of the mother (“relative neonatal body mass”, RNBM) varies immensely across mammals; it ranges from less than a percent in most bears up to almost 50% in some bats. Neonatal body mass scales negatively with respect to maternal body mass (Figure 4; allometric exponent of 0.93, differing from 1 at *P* < .001). Relative neonatal body mass thus decreases with female mass, a pattern also found in earlier studies and hypothesized already by D'Arcy Thompson (Leitch, Hytten, & Billewicz, 1959; see Leutenegger, 1976 for a discussion of allometric scaling exponents of neonatal vs maternal weight). We were unable to account for phylogeny here because mammalian phylogenetic relationships remain unresolved, especially with regard to the position of several major groups like the bats (Chiroptera) and ungulates (Cetartiodactyla and Perissodactyla) (eg, Esselstyn, Oliveros, Swanson, & Faircloth, 2017; Meredith et al., 2011; O'Leary et al., 2013; Tarver et al., 2016; see also Foley, Springer, & Teeling, 2016). Moreover, insufficient genetic data are currently available for all 284 species used in our analysis to construct a well‐supported phylogenetic tree without substantially reducing our sample size.

Note that humans, despite having perhaps the hardest births among mammals, do not have the largest neonates. In fact, bats exhibit the highest values of relative neonatal body mass, both within their weight classes and overall (Figure 4). They also exhibit a particular pelvic sexual dimorphism, which makes them an interesting study taxon for human obstetrics (discussed below). Also, all aquatic mammals show a higher RNBM within their size class than humans. This is true for the only two fully aquatic mammalian taxa, whales (Cetacea) and sea cows (Sirenia), but also for highly aquatic subtaxa of otherwise terrestrial taxa: within the Carnivora, seals (Pinnipedia) show a higher relative neonatal body mass than the non‐aquatic carnivorans (“Fissipedia“), as has been shown previously (eg, Webb, 1997). The small relative neonatal size of terrestrial carnivorans (“Fissipedia”) is exemplified by bears or big cats with RNBM values of about 1% or lower (see *Ursus* and *Panthera* in Table S1). An exception is the most aquatic terrestrial carnivoran, the sea otter (*Enhydra lutris*, Table S1). Although neonatal size is subject to various selection pressures and is linked to other aspects of life history (eg, gestation length and litter size), the comparatively large neonatal size of aquatic mammals may in part be an adaptation to thermoregulation (heat dissipation in water is higher than on land) and, in the case of the fully aquatic whales and sea cows, also due to the fact that neonates must be able to swim and follow the mother immediately (Webb, 1997). This kind of “forced precociality,” and hence relatively large neonatal body mass, can also be seen in many cursorial ungulates whose neonates need to follow their mother or the herd immediately after parturition (eg, the reindeer *Rangifer tarandus*, zebras *Equus* spp., and different taxa of bovid, for example, *Syncerus*, *Tragelaphus*, *Nanger*, or *Antidorcas;* see Table S1). Future study, including improved phylogenetic information, will have to elucidate to what extent variation in mammalian relative neonatal body size reflects adaptive variation.

The large neonatal size of aquatic mammals is also interesting in the context of the pelvic floor hypothesis. In the water, the weight of the animal is offset by buoyancy, and the pressure of the viscera on the pelvic floor is counteracted by the water. Cetacea (whales and dolphins) only have a rudimentary pelvis that does not constrain the passage of the fetus. Seals, however, have a complete and fully functional pelvic girdle, which articulates with the hindlimbs. Yet, seals have very large neonates. For example, northern fur seals and harbor seals have a body mass comparable to that of humans, but they have neonates twice as heavy as in humans (an RNBM of 13.0% and 13.4%, respectively; Tague, 2016; Table S1). Indeed, the pinniped pubic symphysis is unfused (Berta, Sumich, & Kovacs, 2015); the innominate bones are connected by a ligament affording some degree of flexibility, in contrast to the terrestrial carnivoran (“fissiped”) condition (see below). Of course, seals have smaller brains than humans and are less orthograde, but the relaxed demands on their pelvic floor as a result of their highly aquatic lifestyle may afford them a more spacious and flexible birth canal through which to pass the fetus.

**Figure 5:**
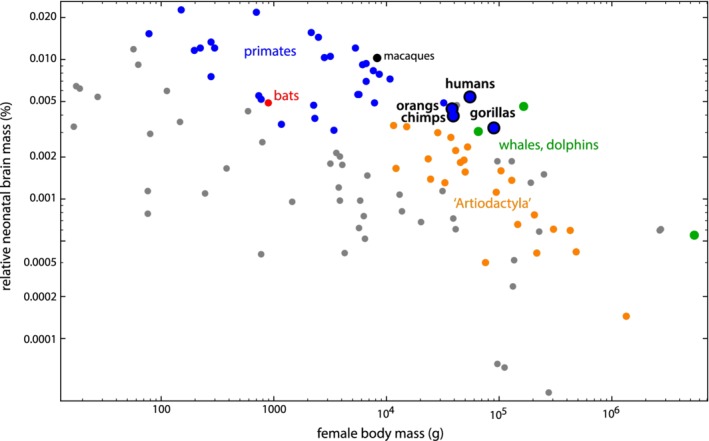
Relative neonatal brain mass (neonatal brain mass as percentage of maternal body mass) versus female body mass for 109 mammalian species on a log‐log scale (data from Capellini et al., 2010a, 2010b). For an explanation of ‘Artiodactyla’, see caption of Figure 4. For the number of species by higher‐order taxon, see Table S2

While bats, seals, and even most ungulates (including cetaceans) all have larger relative neonatal body mass than humans, neonatal *brain* mass—as percentage of maternal body mass—is highest in primates (Figure 5). Within their body size class, humans, macaques, but also whales and dolphins have exceptionally large neonatal brains. Unfortunately, our data comprise only a single bat species (Indian flying fox, *Pteropus giganteus*, one of the largest bats), which has a relative neonatal brain mass similar to that of humans. Within its size class, however, its relative neonatal brain mass is not exceptionally high. Many other bats, however, especially smaller‐bodied species, have larger adult brains relative to adult body weight (Baron, Stephan, & Frahm, 1996) than the bat species in Capellini et al.’s data, and thus likely also larger neonatal brain sizes.

**Figure 6:**
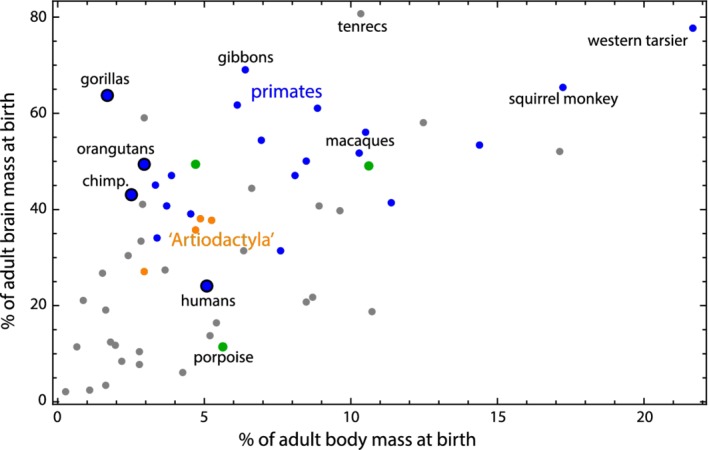
Percentage of adult brain mass achieved at birth versus percentage of adult body mass achieved at birth for 59 mammalian species on a log‐log scale (the species covered both by Boddy et al., 2012a, 2012b, and by Capellini et al., 2010a, 2010b). This plot represents an altriciality‐precociality axis separately for the entire body and the brain. For an explanation of ‘Artiodactyla’, see caption of Figure 4. For the number of species by higher‐order taxon, see Table S2

The degree of developmental maturity at birth is one of the most important life history parameters affecting neonatal body mass (Martin & MacLarnon, 1985). It is commonly claimed that the evolutionary increase of brain size in the human lineage was paralleled by secondary altriciality in order to ease childbirth (Montagu, 1961; Portmann, 1941; Rosenberg & Trevathan, 2002). In Figure 6, we plotted the percentage of adult body mass achieved at birth against the percentage of adult brain mass achieved at birth. These two variables estimate the degree of developmental maturity at birth separately for the entire body and for the brain. On average, humans have achieved 5% of their total adult body mass at birth, and already 24% of their adult brain mass. This means that humans are about as developed as the other great apes and lar gibbons concerning body mass, but far more altricial regarding brain mass (compared to 40%‐70% in the apes). On the other side of the spectrum are tarsiers (~80% of brain mass and 22% of body mass) and squirrel monkeys (~65% of brain mass and 17% of body mass) with precocial newborns (Figure 6).

## 5 SEXUAL DIMORPHISM: A COMPARISON WITHIN SPECIES

Most current explanations of the high rates of cephalopelvic disproportion in humans assume a trade‐off between obstetric selection toward a large birth canal and a counteracting selection that favors a narrower pelvis. The hypotheses differ in the proposed source of this counteracting selection that limits an evolutionary widening of the birth canal. These selective forces likely arose in two different bouts: early selection incurred by the requirements of bipedal gait or visceral support, and a later selective phase associated with both carrying (requiring extended visceral support) and giving birth to a large fetus. While the first selective bout affected both sexes, the latter one was limited to women and likely gave rise to the sexual dimorphism of pelvic form observable today. Disentangling pelvic features that differ between species in both sexes, versus features that differ between the sexes of a single species, offers a way to study the selective regimes and evolutionary responses of the two phases.

The well‐studied sexual dimorphism in the human pelvis, specifically in the spaciousness of the birth canal, is also seen in other primate species with high cephalopelvic indices, suggesting that this dimorphism evolved as a consequence of the increase in relative fetal size (Moffett, 2017; Zollikofer, Scherrer, & Ponce de León, 2017). To appreciate the magnitude of sexual dimorphism, it is crucial to emphasize the specific conditions required for sexually dimorphic characters to arise in evolution. Most importantly, sexual dimorphism in traits that are present in both sexes and underlain by the same genetic factors requires divergent selection in both sexes; directional selection in one sex alone is not sufficient (Lande, 1980). Selection in one sex, *without antagonistic selection in the other sex*, usually leads to correlated response in another sex, given that the genetic‐developmental basis of the trait in males and females is the same (ie, there is a high genetic correlation between male and female traits). The fact that males nevertheless maintained a narrow pelvis implies that *there has been selection against the widening of the pelvis in males*.

Two different selection regimes are conceivable: First, males and females could share the same selection pressure for a narrow pelvis (as is implied for gait and visceral support), which is opposed by obstetric selection only in females. In this case, the shared selection can be studied directly in males, without the need to account for obstetric demands. Second, the selection in males may result from a male‐specific function. In this case, it can impose selection for a narrow pelvis in females to the extent that the pelves of the two sexes are correlated.

We thus ask if there are advantages of a narrow pelvis specific to males. Given that a broader pelvis may be associated with a weaker or more vulnerable pelvic floor also in males, the medical literature on disorders of the male pelvic floor offers interesting insights. The pelvic floor in males is involved in sphincter functions as in females, but is also crucial in sexual performance, specifically in maintaining erection. Disorders of male pelvic floor functions include various effects on urinary and fecal continence, rectal prolapse (which is rare in men), and on overall well‐being (eg, pelvic pain, which is common in men). A further well‐documented medical condition in males is erectile dysfunction, including orgasmic or ejaculatory dysfunction (Cohen, Gonzalez, & Goldstein, 2016), which may be a consequence of a weak pelvic floor. This reasoning has an evolutionary component. Human males differ from other primates and many other mammals in that they possess a large pendulous penis, which, at the same time, lacks a penile bone, the baculum. The size of the baculum among primates correlates positively with body size and is generally greater in species with long intromission (Dixson, 2012). Human males score high on both characteristics, yet lack a penile bone. This may impose additional requirements on the human pelvic floor, because the muscles of the pelvic floor and the penile basis, in particular the ischiocavernosus and bulbospongiosus (bulbocavernosus) muscles, act as a part of the complex involuntary muscular interplay establishing and maintaining the pressure during erection and contributing to ejaculation. It is not known exactly when the baculum was lost in the human lineage, or whether perhaps a pelvis that was already narrow enabled its loss. In any case, erectile function may be influenced in part by pelvic floor strength and thus also by pelvic anatomy.

To our knowledge, the existing literature does not include direct estimates of how frequently a weak pelvic floor is the main cause of erectile dysfunction. However, many recent studies have shown that improvement of the strength of the pelvic floor considerably alleviates erectile problems in a substantial percentage of men (eg, Cohen et al., 2016). For example, a study conducted on a small group of men (*N* = 55) has shown large improvement of erectile function after 6 months of exercise of the pelvic floor muscles (with 40% regaining normal erectile function, and a further 35.5% showing improvement; Dorey, Speakman, Feneley, Swinkels, & Dunn, 2005). Further studies, including large randomized trials, consistently show considerable positive effects of exercise on erectile dysfunction (Lavoisier et al., 2014; Prota et al., 2012; Siegel, 2014). The incidence of erectile dysfunction varies across studies and populations, and it is strongly age‐dependent, but all studies agree that it is a common phenomenon. The large Massachusetts Male Aging Study (Feldman et al., 2000; Johannes et al., 2000; Travison et al., 2007) reports severe erectile dysfunction to affect 5%‐10% of men below 40 and 10% of men aged 40‐70, with another 25% experiencing intermittent erectile difficulties. Whereas vascular diseases and aging are the best‐documented and often the most immediate triggers of erectile dysfunction, such proximate factors do not exclude the possibility that men with an anatomically weaker pelvic floor are more likely to develop erectile dysfunction.

Taken together, pelvic sexual dimorphism evidences selection for a narrow bony pelvis in men, and medical data suggest that a weak pelvic floor may affect male reproduction via its effect on erectile function. The currently missing piece of information is whether the narrow bony pelvis is indeed associated with a stronger pelvic floor in human males. We are not aware of studies that have directly addressed this question in men, but several studies showed a correlation between pelvic floor disorders and pelvic width in women (Brown et al., 2012; Handa et al., 2003; Sze et al., 1999).

Interestingly, bony pelvic morphology has been associated with penile morphology in cetaceans (whales and dolphins). Dines et al. (2014) found that in species with intense sexual selection, males had larger penises and larger pelvic bones relative to their body size than males of species with less intense sexual selection. The pelvic bones serve to anchor the ischiocavernosus muscles in cetaceans (Slijper, 1966), which in turn maneuver the penis and appear important in maintaining erection (Ommanney, 1932). Cetaceans, like humans, lack a baculum (Slijper, 1966), a situation that may require greater pelvic support. It thus appears, that the pelvic bones of cetaceans serve an important function in reproductive behavior (rather than being entirely vestigial) and have responded to selection for penile and erectile function (Dines et al., 2014).

## 6 MAMMALIAN PUBIC SYMPHYSIS MORPHOLOGY

The human pubic symphysis is a joint with limited flexibility, capable of a small amount of movement in adults. It consists of a fibrocartilaginous disc filling the gap between the two pubic bones (Rosse & Gaddum‐Rosse, 1997). The joint is robust and highly resistant to shearing and compression (Becker, Woodley, & Stringer, 2010), but rupture can occur during childbirth (Boland, 1933). Despite being very robust, the pubic symphysis is able to widen and increase in flexibility in human pregnancy under hormonal influence. By widening the symphysis before parturition, the birth canal gains flexibility without modifying the dimensions of the bony pelvis.

The flexibility of the pubic symphysis may be considered an evolutionary adaptation to ease the difficulty of human childbirth. In several other species, however, flexibility of the pubic symphysis is much larger than in humans. This pattern is seen in different mammalian species that have to accommodate large neonates through a narrow birth canal. Guinea pigs, for example, face the extreme situation where the mean diameter of the fetal head is 20 mm, whereas the pelvic canal in early pregnancy is just 11 mm wide (Ruth, 1937). To accommodate the fetal head, the pubic bones separate up to 23 mm at the pubic symphysis in female guinea pigs during late pregnancy and parturition (Todd, 1923), and a ligament appears in the middle of the joint. At the symphyseal surfaces of the pubic bones, bone resorption occurs (Ortega, Muñoz‐de‐Toro, Luque, & Montes, 2003). The modifications of the pubic symphysis involve hormonally regulated adaptations of the connective tissue mediated by estrogen and relaxin during pregnancy (Schwabe & Büllesbach, 1990). The resulting increased mobility in the pubic symphysis is a prerequisite for successful delivery in guinea pigs.

The appearance of an interpubic gap bridged by a flexible ligament (ie, the absence of a true symphysis) is not exclusive to guinea pigs, but also occurs in mice, bats, deer mice, macaques, and humans (Schwabe et al., 1978; Todd, 1921), to name a few. In mice, the gap measures 4‐10 mm at delivery (Ortega et al., 2003). In humans, pubic symphyseal flexibility is more limited. The mean increase in width of the interpubic gap has been estimated to be 3 mm (Becker et al., 2010; Hisaw & Zarrow, 1950). Hisaw (1924, 1925) reported that in the pocket gopher (*Geomys bursarius*), a fossorial rodent, the pelvis forms a complete ring in both sexes, but in females the pubes begin to resorb in the first breeding season, with a complete “pubiolysis” by the time of copulation. The female then keeps her open pelvis for the rest of her life. Which parts of the birth canal are relaxed also differs between species. In humans, the sacroiliac joint relaxes in addition to the pubic symphysis under the influence of relaxin, whereas in guinea pigs remodeling is limited to the pubic symphysis alone (Schwabe & Büllesbach, 1990).

In some other mammals, for example in many odd‐ and even‐toed ungulates and in big cats, the pubic symphysis is fused by ossification by the time animals reach adulthood and represents the morphological condition throughout the entire reproductive career (Todd, 1921; Figure 2 and Figure S11). The consequential inflexibility of the joint in these species likely results in a higher net force applied by the muscles to the rest of the body and thus facilitates more energetically efficient locomotion. Even though the neonates in these species can be large (eg, Cetartiodactyla), they have relatively much smaller heads than human neonates (Figure 5).

From this comparative perspective, the question arises why the human pubic symphysis is so *in*flexible despite the strong obstetric burden. The medical literature provides one (mechanistic) explanation for the lack of larger symphyseal flexibility: great symphyseal width in humans during pregnancy and birth is associated with severe pelvic girdle pain (Björklund, Bergström, Nordström, & Ulmsten, 2000; Björklund, Nordström, & Bergström, 1999), which is a poorly understood condition but common among athletes, patients with traumatic pelvic injuries, and pregnant women (Becker et al., 2010; Cheer & Pearce, 2009; Ronchetti, Vleeming, & van Wingerden, 2008). It is aggravated by weight‐bearing and associated with difficulty in walking (Jain, Eedarapalli, Jamjute, & Sawdy, 2006). As the medical literature also indicates higher rates of prolapse and incontinence in women with a wider pelvis (Brown et al., 2012; Handa et al., 2003; Sze et al., 1999), a greater symphyseal flexibility may also weaken the pelvic floor. Therefore, with our mode of locomotion, substantial widening of the symphysis may not be viable due to the reduced stability of the pelvis and the pelvic floor.

## 7 BATS AND THE PELVIC FLOOR HYPOTHESIS

Bats (Mammalia: Chiroptera) are a taxonomically rich clade of mammals, with species weighing between 2 g, as in *Craseonycteris thonglongyai* (Hill & Smith, 1981), up to approximately 1 kg, as in *Acerodon jubatus* and *Pteropus vampyrus lanensis* (Stier & Mildenstein, 2005). As the only mammals that have evolved powered flight, their postcranial skeleton has undergone several evolutionary modifications to accommodate active aerial locomotion (Neuweiler, 2000). Most of these anatomical adaptations are found in the forelimb, which constitutes the major portion of the wing and the pectoral girdle, and reflect the trade‐offs between adequate structural support, elasticity, and reduction of weight (Adams & Thibault, 1999; Swartz & Middleton, 2008). The functionality of the chiropteran hindlimb for terrestrial locomotion is reduced compared with other terrestrial mammals. The hindlimbs in bats are functionally adapted to serve as hooks to attach to the mother from birth, and to the roost surface (Reyes‐Amaya, Jerez, & Flores, 2017). All bat species show “hindlimb reversion”, where the femora extend laterally or caudally and are rotated so that the knees point dorsally, with the plantar surfaces (soles) of the hind feet facing ventrally. It permits the claws to grip when a bat hangs head‐down with its chest against a surface. Depending on the species' capabilities for terrestrial locomotion, the hip joints are rotated to different degrees (Riskin, Bertram, & Hermanson, 2016; Vaughan, 1970), up to almost 180° in Phyllostomatidae and Natalidae, the least capable of crawling. The acetabulum is wider (presumably allowing a greater range of femoral motion) and the head of the femur is offset from the long axis of the bone to a greater degree in bats that are proficient at terrestrial locomotion than in less proficiently crawling species (Dwyer, 1960, 1962; Riskin et al., 2016; Vaughan, 1970).

Apart from powered flight, another striking feature of bats is that they all give birth to very large neonates relative to maternal size (Figure 4) (Badwaik & Rasweiler, 2000; Hayssen & Kunz, 1996; Kunz & Kurta, 1987). The relatively largest neonates reach a mass of around 45% of maternal body mass (as in *Rhinolophus cornutus* and *Anoura geoffroyi*; Hayssen, Van Tienhoven, & Van Tienhoven, 1993; Kulzer, 2005; Kunz & Kurta, 1987), while the relatively smallest neonates still make up at least 10% of maternal mass (as in *Pteropus poliocephalus* and *Taphozous longimanus*; ibid.). The wide range in relative neonatal body mass in bats partly owes to differences in adult body size. The larger‐bodied Old World fruit bats (Pteropodidae) tend to give birth to relatively small neonates, whereas some of the smallest‐bodied bats (eg, Phyllostomidae and Rhinolophidae) have the relatively largest neonates (Hayssen & Kunz, 1996; Kunz & Hood, 2000; Kunz & Kurta, 1987; Reiter, 2004). Relative neonatal size thus also shows a phylogenetic signal (Badwaik & Rasweiler, 2000; Kunz & Kurta, 1987). Most bat species have litters of a single pup, although some species tend to give birth to twins (Vespertilionidae). In the latter case, the individual neonates tend to be smaller than those of related species that have a litter size of one (Badwaik & Rasweiler, 2000; Koehler & Barclay, 2000).

Bats have by far the largest neonates for their body size, even when compared to non‐volant (ie, non‐flying) mammals of similar size (Figure 4 and Table S1) (Kunz & Hood, 2000; Kunz & Kurta, 1987). Bats have a unique combination of life history traits, sharing characteristics like longevity and reproductive strategies with large mammals, and characteristics like fast prenatal and postnatal growth with (other) small mammals. Small mammals (<3 kg) tend to have fast life histories and are *r*‐selected. They have a fast prenatal and postnatal development, short lifespans and interbirth intervals, along with large litters consisting of multiple small neonates (eg, Jones et al., 2009). Larger mammals tend to have slower life histories (*K*‐selected). Bats are not typical small mammals in this regard. In fact, they almost seem like large mammals that have to be small in order to fly. The median adult weight of chiropterans is around 20 g (Jones et al., 2009), but their gestation length is approximately three to four times longer than those of other placental mammals of comparable body size. For species with an adult body mass below 20 g, mean gestation length is 102 days for bats compared to 25 days for non‐chiropteran placentals; for species with an adult body mass above 20 g, mean gestation length is 124 days for bats compared to 45 days for non‐chiropterans (data from Jones et al., 2009). Bats also most often bear a single, large pup only once or twice a year, and they can live up to 41 years (*Myotis brandtii*) (Podlutsky, Khritankov, Ovodov, & Austad, 2005). The great relative size of their neonates is not the only way in which female bats disproportionately invest in their offspring; bats are also the only mammals to suckle their young until they have nearly reached adult size (Kunz, 1987). As bats are specialized for flight, they are vulnerable during the time their wings are not fully developed. This likely accounts for why their postnatal development is fast, as is typical of small mammals. Bats are born with a full set of deciduous teeth and well‐developed thumbs and feet, with mostly ossified bones and prominent claws, so that they can either cling to the mother, which cannot actively hold her pup during flight, or to the roosting site (Badwaik & Rasweiler, 2000). A large neonatal body size is also likely required for thermoregulation (Badwaik & Rasweiler, 2000; Kunz & Hood, 2000; Kunz & Kurta, 1987).

Pelvic morphology in bats is strongly sexually dimorphic (Chapman, Hall, & Bennett, 1994; Crelin, 1969; Crelin & Newton, 1969; Ekeolu & Ozegbe, 2012; Hamre, Meyer, & Martin, 1928; Nwoha, 2000; Todd, 1921; Walton & Walton, 1968). In adult males of most species, the pubic bones form a bony ring (or bar) ventrally, with the left and right pubic bones fused by a synostosis (Crelin & Newton, 1969; Nwoha, 2000; see also Figure 2C,D and Figures S2‐S7). The pubic bones in the adult female pelvis, conversely, do not meet but rather create a pubic gap that is bridged medially by an interpubic ligament consisting of fibrocartilage (Crelin, 1969; Nwoha, 2000; O'Connor, Cain, & Zarrow, 1966; Nwoha, 2000; Figure 2C,D and Figures S1‐S7). Although the width of the pubic gap varies considerably across chiropteran species, female bats systematically lack a true pubic symphysis, whereas adult male bats nearly always exhibit a synostosis (Hamre et al., 1928; Nwoha, 2000; O'Connor et al., 1966; NDSG observations). The histology of the pubic symphysis and the dimensions of the fibrocartilaginous ligament in female bats have been shown to change throughout both ontogeny and pregnancy (Crelin, 1969; Crelin & Newton, 1969; Hamre et al., 1928; O'Connor et al., 1966).

This ligament, which can span up to the maximum transverse diameter of the pelvic canal (or beyond!) in some species (eg, *Rousettus aegyptiacus*, *Glossophaga soricina, Pteropus* spp.; see Figure 2D and Figures S1 and S7), enables female bats to deliver their large neonates through a pelvic canal that would otherwise be too small (Crelin, 1969). The size of the male pelvis, with its lack of mobility due to the fusion of the pubic symphysis (including the sacro‐iliac joint in some species) would not be able to accommodate neonates that are commonly 20%‐40% of adult female body size. This strong sexual dimorphism in the pelvis documents the divergent selection regimes in males and females (Lande, 1980; and discussed above).

Although bats are not the only mammals with the pubic bones separated by a large ligament in females (which can create the illusion of an “open pelvis” in osteological specimens), they are the only major mammalian group in which all its members possess this pattern of sexual dimorphism in pelvic morphology. How did bats manage to evolve an “open” pelvis, when the vast majority of mammals did not, including humans and a few other primates that clearly could have benefited from a more mobile and elastic pubic symphysis for the purpose of parturition? Apart from the relatively small bats, an open pelvis is only observed in small non‐volant mammals (eg, hedgehogs, a few rodents, tenrecs; Figure 2E and Figures S8, S9, and S16), suggesting that overall body size might constrain the width of the pubic aperture due to the positive allometric scaling of organ weight relative to pelvic floor area and muscle attachment area on the weight‐bearing bones (Christiansen, 2002).

As the aforementioned small non‐volant mammals with a large interpubic ligament all have litters of many relatively small young, we propose that their pelvic morphology may not have been selected for childbirth but for affording females more space in the abdominal cavity to gestate multiple fetuses (eg, in tenrecs and hedgehogs). In bats, by contrast, we suggest that it is the delivery of the (usually) single large neonate that selected for a more capacious pelvis, including the birth canal. This is corroborated by the large pubic aperture found in pteropodids, the largest‐bodied bats: If space in the abdominal cavity was the relevant constraint also in bats, large‐bodied bats (with sufficient abdominal space to accommodate a single fetus) would not exhibit a large pubic aperture (eg, Figure 2D and Figure S7).

We postulate that two factors have allowed female bats to widen and open up their pelvis ventrally: (1) the reduced role of the hind limbs in supporting the body, and (2) the large reduction of pressure on the pelvic floor as a consequence of their roosting behavior. In addition to having powered flight as their main mode of locomotion, which imposes the strongest mechanical demands on the forelimbs instead of the hind limbs, many bat species spend a considerable portion of every day (up to 12 hours or longer; Altringham, 1999; Hamilton & Barclay, 1994) roosting head down, with gravity acting in the direction opposite to the pelvic floor (Figure 1C). This unique feature of bat roosting behavior makes bats an interesting case study to test the pelvic floor hypothesis. In contrast to erect humans, where the center of mass is directly located above the pelvis, bats may experience comparatively little and infrequent pressure of the viscera and the fetus on their pelvic floor.

We predict that the length of the interpubic ligament in bats, represented by the width of the ventral pubic aperture in females, is correlated with relative neonatal body mass, but data to test this hypothesis are currently not available. Not all bats roost head down, however. Some species are crevice‐dwellers, bats that crawl into very small openings in rock faces, trees, human constructions, etc. (Boonman, 2000; Russo, Cistrone, Jones, & Mazzoleni, 2004). In these confined spaces, bats are not always positioned head down (Altringham, 1999; Kunz, 1982). Furthermore, there are two small groups of distantly related bats that roost in upright positions, hanging from adhesive pads on their thumbs or wrists. These two groups, the Thyropteridae and Myzopodidae, evolved this adaptation independently (Goodman, Rakotondraparany, & Kofoky, 2007; Riskin & Racey, 2010; Velazco, Gregorin, Voss, & Simmons, 2014). The roosting behavior of bats and the associated variation in body orientations enables a test of the pelvic floor hypothesis: do head‐down‐roosting bat species have a proportionately larger pubic aperture relative to the size of their neonates than upright roosting and crevice‐dwelling bats?

A further way of testing the pelvic floor hypothesis is to compare bats with different flying modes. Acrobatic flyers with high maneuverability in order to catch flying insects are likely to incur more impact and pressure on their pelvic floor through forces of acceleration (sudden changes in speed or direction) than, for example, frugivorous and nectarivorous bats that do not hunt in flight. The former (see Figures S2, S3, and S5) might therefore have narrower pelvic outlets and smaller interpubic gaps (in females) than the latter (Figure 2D and S1 and S7) as an adaptation for a strong pelvic floor. Pelvic shape might thus also be correlated with certain traits connected to flight mode, such as wing loading and aspect ratio (a wing shape parameter). Data on the type of flight, wing loading and aspect ratio are available for many species (eg, Aldridge & Rautenbach, 1987; Dietz, von Helversen, & Nill, 2007; Müller et al., 2012; Norberg & Rayner, 1987), but have never been analyzed in this context.

## 8 CONCLUSIONS AND OUTLOOK

Deciphering the many selective pressures and constraints that have shaped the evolution of the human pelvis—including locomotion, the weight‐bearing support function, and childbirth—is highly challenging, especially within samples of only modern humans. For many decades, Washburn's (1960) “obstetrical dilemma” had remained untested, and recent attempts are not particularly conclusive. Apart from clinical studies showing an association of pelvic floor disorders and pelvic width, Abitbol's (1988) pelvic floor hypothesis remained untested as well. Climatic adaptation of human body proportions, including bi‐iliac breadth, is well documented, but the degree to which this affects birth relevant dimensions is less well known. Here, we aimed to show that a comparative study of hard and soft tissue anatomy in combination with reproductive ecology in mammals enables the testing of existing evolutionary hypotheses on human childbirth and the creation of new ones.

To our knowledge, wide quantitative analyses of pelvic geometry and birth in mammals are not available yet. However, our review of mammalian pelvic form based on qualitative assessments of osteological material and available literature revealed some general patterns. Several small‐bodied species with relatively large neonates tend to show pronounced sexual dimorphism in the pelvis. This is well exemplified in bats and guinea pigs, in which the male condition of a “closed” pelvis would not be able to accommodate the fetus. Even the pelvis in the sexually immature female (as in the pocket gopher and bats) or the female during the early stages of her pregnancy (as in guinea pigs) tends to be too small, which suggests an advantage of a small pelvic canal. Hence, humans are not unique among mammals in having a (too) narrow birth canal. However, these other mammalian species typically undergo a significant “opening” of the pelvis before parturition by widening of the pubic symphysis, which transforms the cartilaginous symphysis into a ligament that affords the flexibility required for giving birth to large neonates. Compared to this, the human symphysis widens only to a very limited extent.

Not all species with a large neonate show such a flexible pelvis, however. Terrestrial even‐toed ungulates (“Artiodactyla”), for instance, have large neonates for their body size (Figure 4) but a closed pelvic canal by means of a fused pubic symphysis both in males and females. Apparently, the biomechanical demands for fast running in these larger‐bodied species have been important for the evolution of their pelvic form. Also, despite a large neonatal body mass, terrestrial ungulates tend to have relatively small neonatal brain masses and thus smaller heads (Figure 5), which eases parturition.

With regard to the classic evolutionary hypotheses about the obstetric conundrum in humans, we suggest that locomotion and posture are differently associated with overall pelvic form in different lineages and environments. For instance, species with very large pelvic canals tend to support their wide, and therefore possibly vulnerable, pelvic floor either by engaging in postures that reduce force on the pelvic floor, or they reduce the pressure of the viscera and the fetus on the pelvic floor by living in water. This supports claims by Abitbol (1988) and others that a major constraint on pelvic width in humans was pelvic floor stability. By contrast, the flexibility of the pelvis, especially of the pubic symphysis, is clearly constrained by functional demands on the pelvis, related to both overall size and locomotion. We thus hypothesize that bipedal locomotion in humans might not primarily have constrained pelvic width—as classically suggested—but the flexibility of the symphysis, which is greatly reduced compared to small mammals with large neonates, but more similar to other large‐bodied bipedal species, such as kangaroos. This is also corroborated by the sexual dimorphism in the human pelvis, which appears small compared to several other mammals with very large neonates, in which females have “open” pelves.

We also briefly discussed possible effects on female anatomy through selection on the male pelvis, but we are not aware of any direct studies of this topic in primates or on a wider mammalian scale. Other, potentially fruitful directions of research include placentation types, which may limit parameters of maternal investment in the fetus, such as gestation length or fetal size, and the anatomy of the cervix, a part of the uterus with a crucial function in pregnancy maintenance and which is highly variable across mammals. Finally, we have closed in on bats, a particularly interesting group with regard to pubic morphology, as a source of insights into the various demands (or lack thereof) on the female pelvis. The variation in bat pelvic morphology, relative neonatal size, and positional behavior makes it possible to study the effect of pelvic floor pressure on pelvic shape while controlling for the confounding effects of locomotion.

## AUTHOR CONTRIBUTIONS

All authors were involved in conceptualizing the ideas and new hypotheses presented in this study. N.D.S.G., F.E.Z., B.F., and P.M. retrieved and/or collated data that was used for analysis in this article. P.M. analyzed the data. N.D.S.G., F.E.Z., M.P., and P.M. drafted the manuscript. All authors contributed to the text and intellectual content of the manuscript and provided critical comments.

## Supporting information

Appendix S1: Supporting information Figures

Table S1 Supporting information Table S1. Data on neonatal and female body mass for 284 mammalian species. Data for 266 species were taken from Tague (2016), and data for an additional twelve species (highlighted in yellow) were collated from the indicated sources. When no reference is given, data are from Tague (2016).

Table S2 Supporting information Table S2. The number of species by higher‐order taxon used to produce each Figure in the main text, including the data source.
