# Supplementary material for: Humans as inverted bats: A comparative approach to the obstetric conundrum
Source: Am J Hum Biol. 2019 Feb 27;31(2):e23227. doi: 10.1002/ajhb.23227 (PMC6492174; doi:10.1002/ajhb.23227)
Supplement: Supplementary file 1 — Appendix S1: Supporting information Figures [file AJHB-31-na-s001.pdf]

Supporting information, Figures S1-S21, Grunstra et al., Humans as inverted bats: A comparative approach to the obstetric conundrum, *American Journal of Human Biology*

Additional photographs of male and female pelves from different mammal species representing all four major branches of placental mammals (Laurasiatheria, Euarchontoglires, Xenarthra, Afrotheria).

Except where single pelves are shown, the male is always on the left and the female on the right side. Unless specified otherwise, pelves are shown in ventral view with the cranial side up. All specimens come from the Mammal Collection of the Natural History Museum Vienna, and collection numbers are given (from left to right).

Size standard: each of the black and white squares has a length of 1 cm.

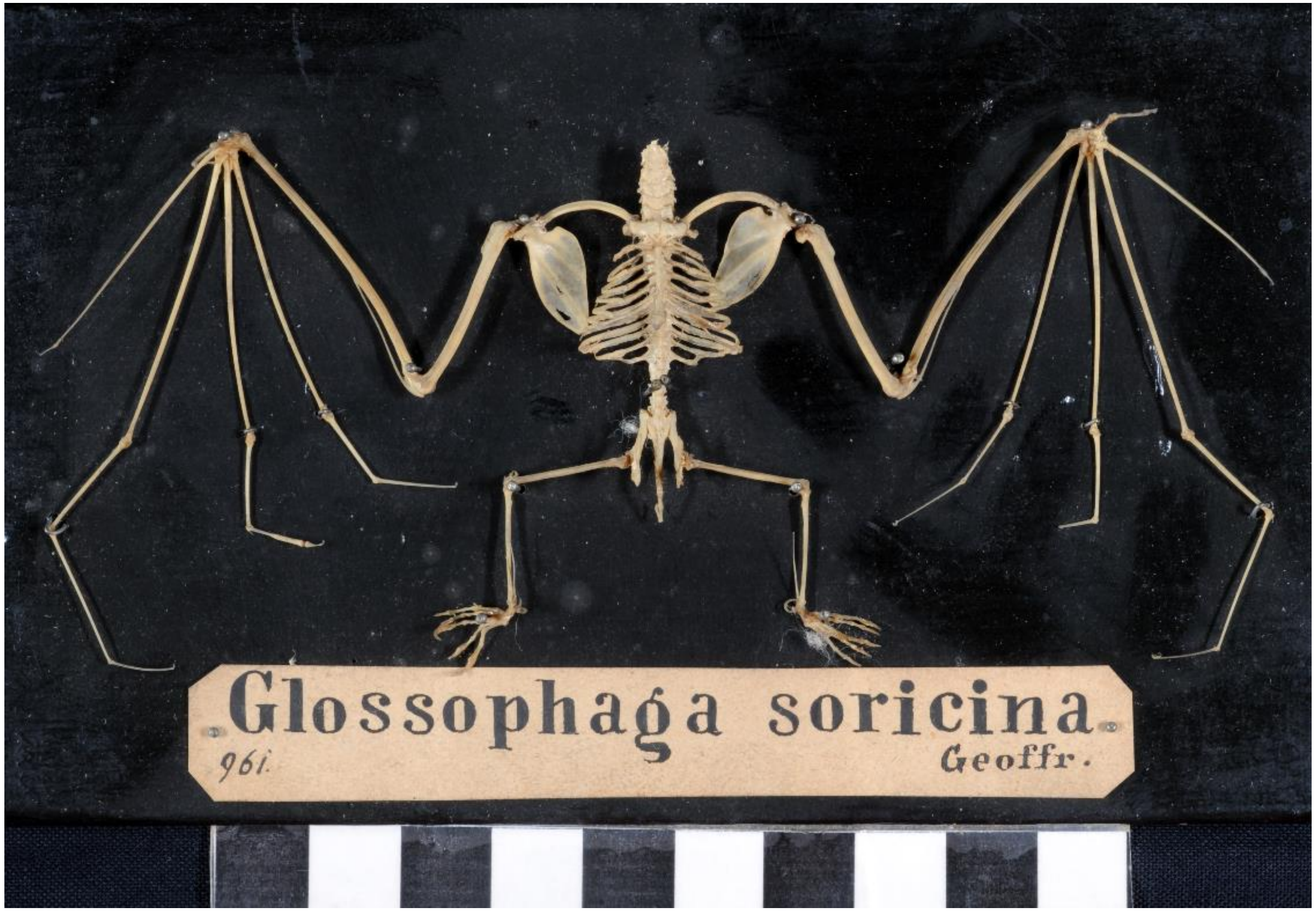

Figure S1. *Glossophaga soricina*, female (Pallas's Longe-tongued bat, Laurasiatheria: Chiroptera). Uncatalogued specimen, no collection number available.

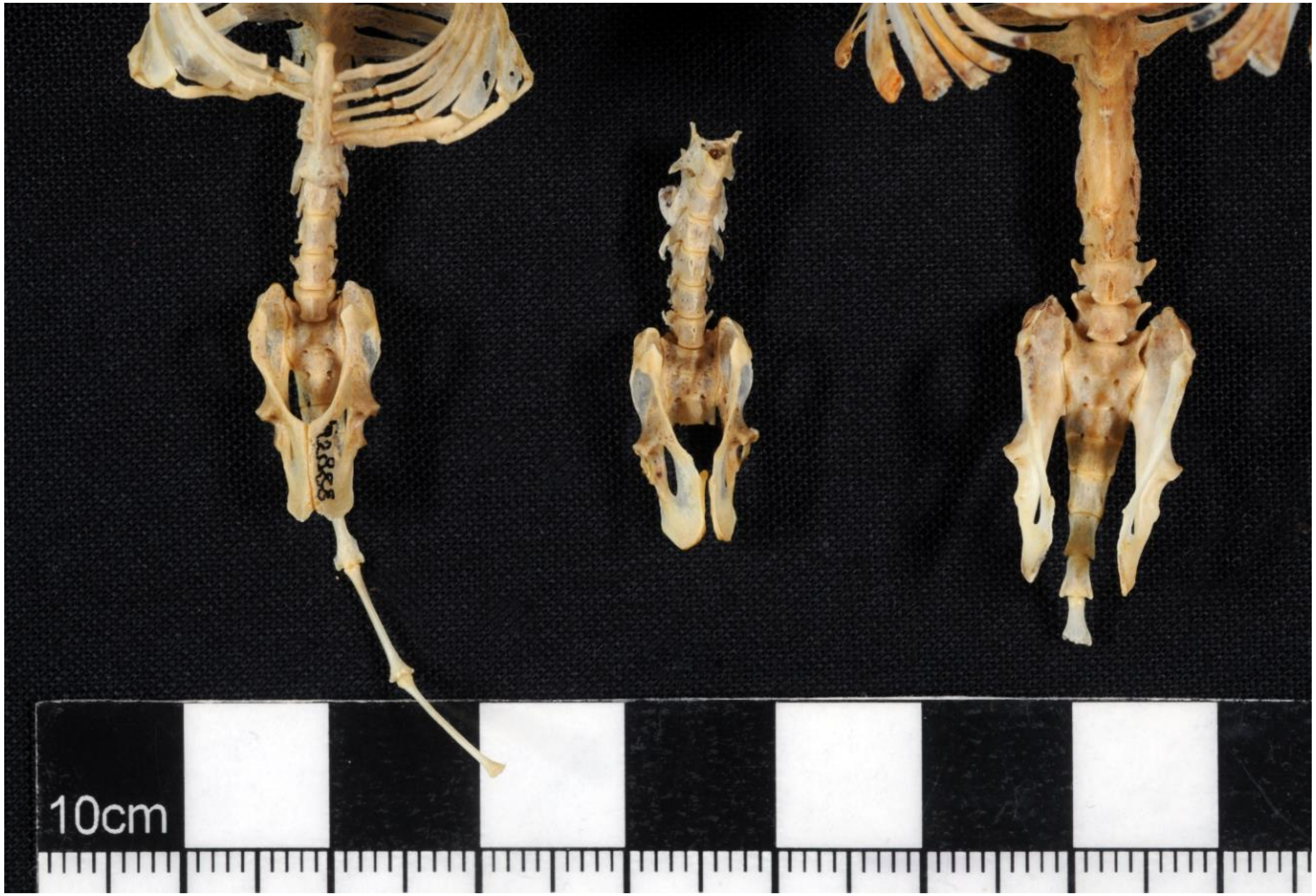

Figure S2. From left to right: *Hipposideros armiger* male (NMW 42888), *H. armiger* female (NMW 42887) (Great roundleaf bat), *H. commersoni* female (NMW 32351) (Commerson's roundleaf bat, Laurasiatheria: Chiroptera).

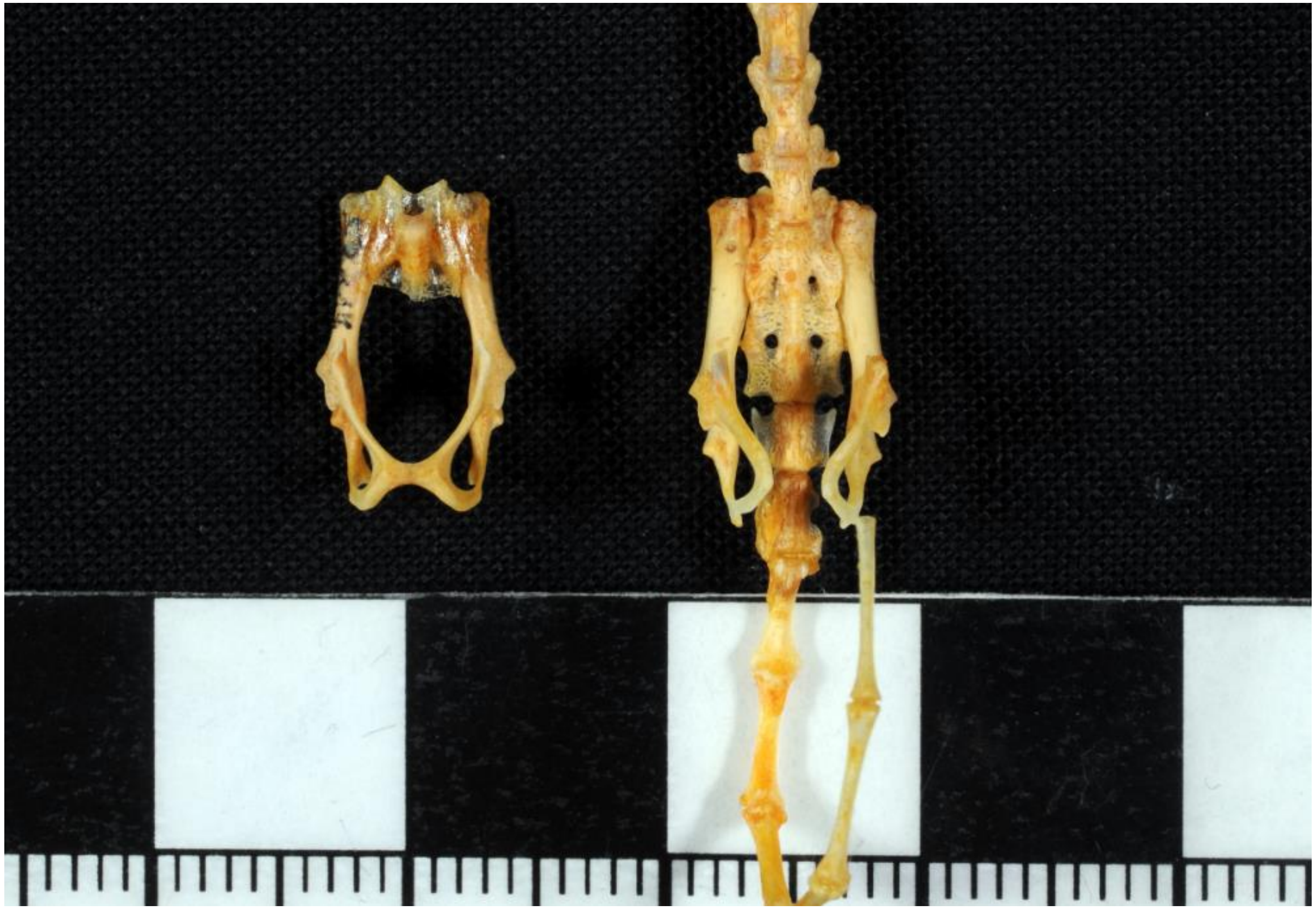

Figure S3. *Eptesicus serotinus* (Serotine bat, Laurasiatheria: Chiroptera). NMW 52817 and NMW 15711.

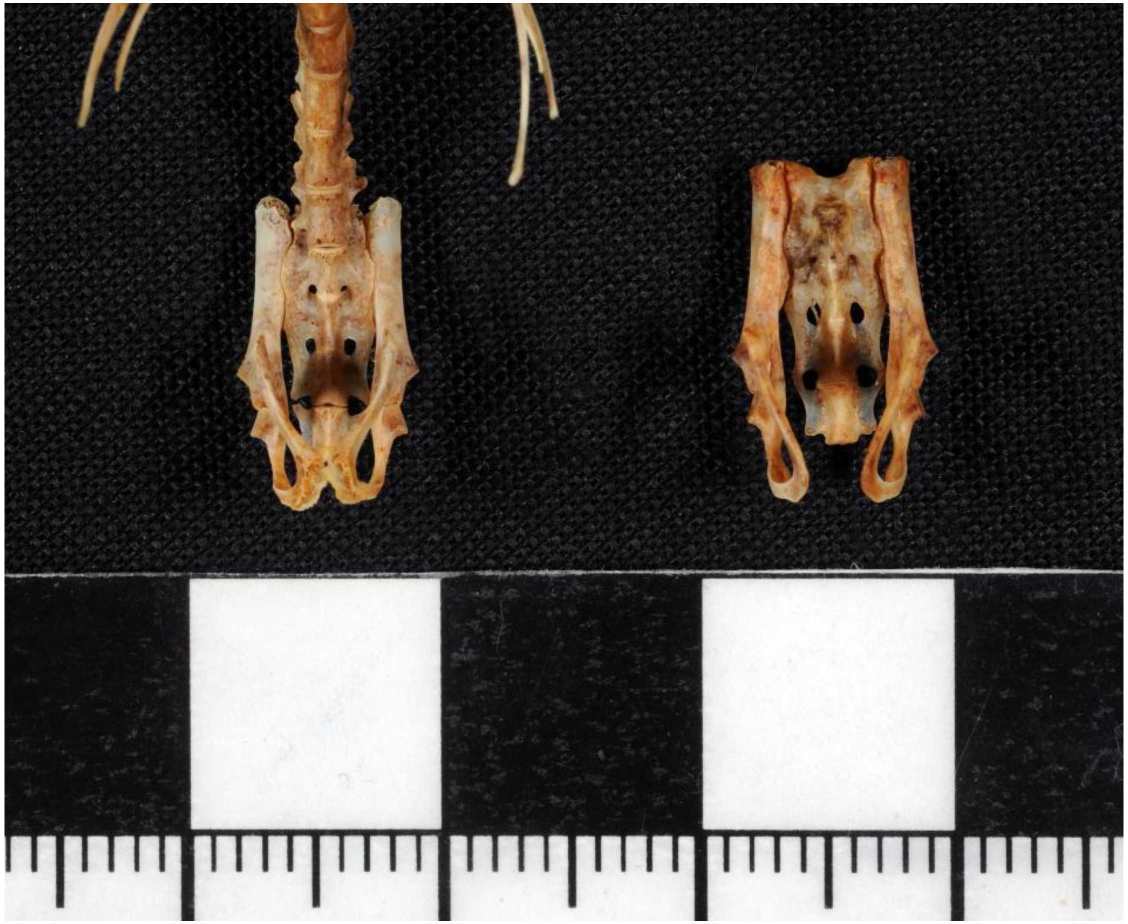

Figure S4. *Myotis myotis* (Greater mouse-eared bat, Laurasiatheria: Chiroptera). NMW 66994 and NMW 66990.

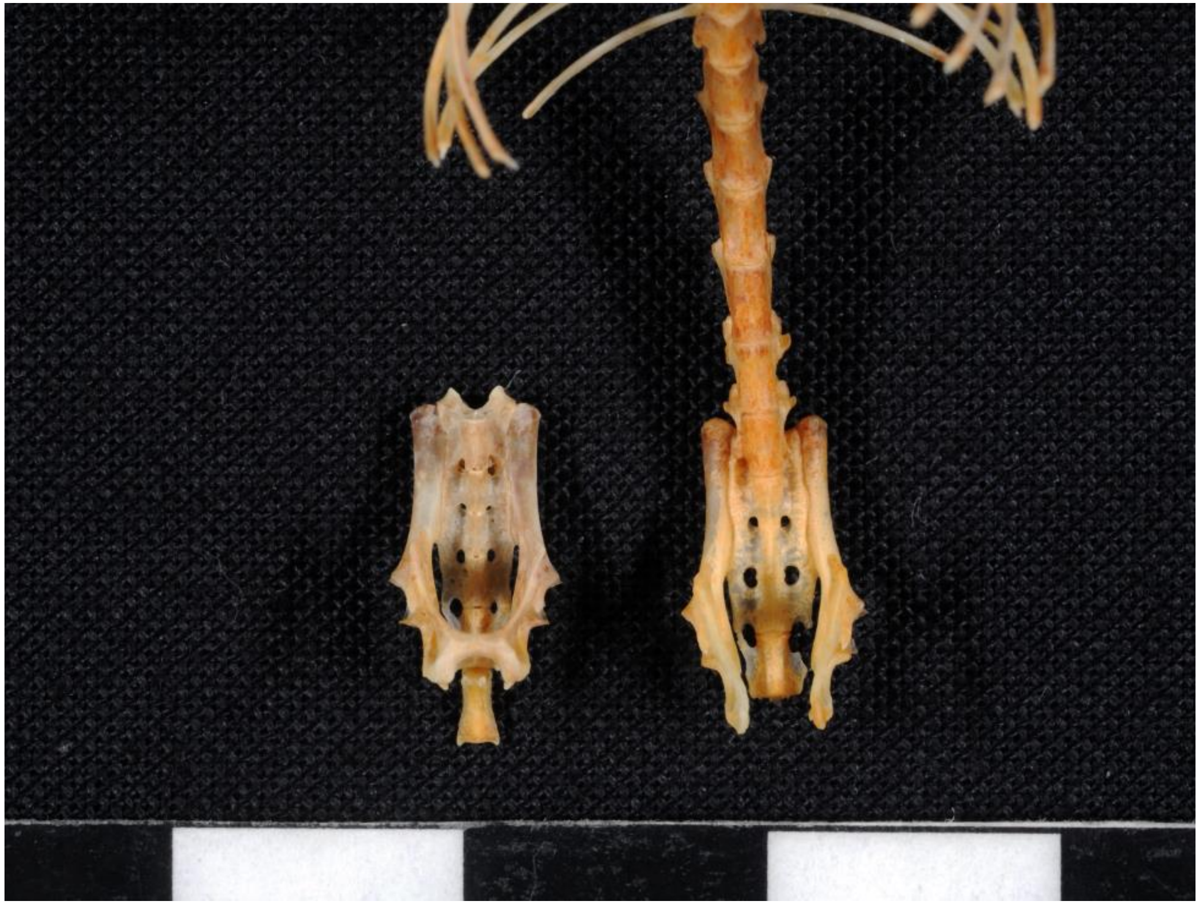

Figure S5. *Vespertilio murinus* (Parti-coloured bat, Laurasiatheria: Chiroptera). NMW 68973 and NMW 68957.

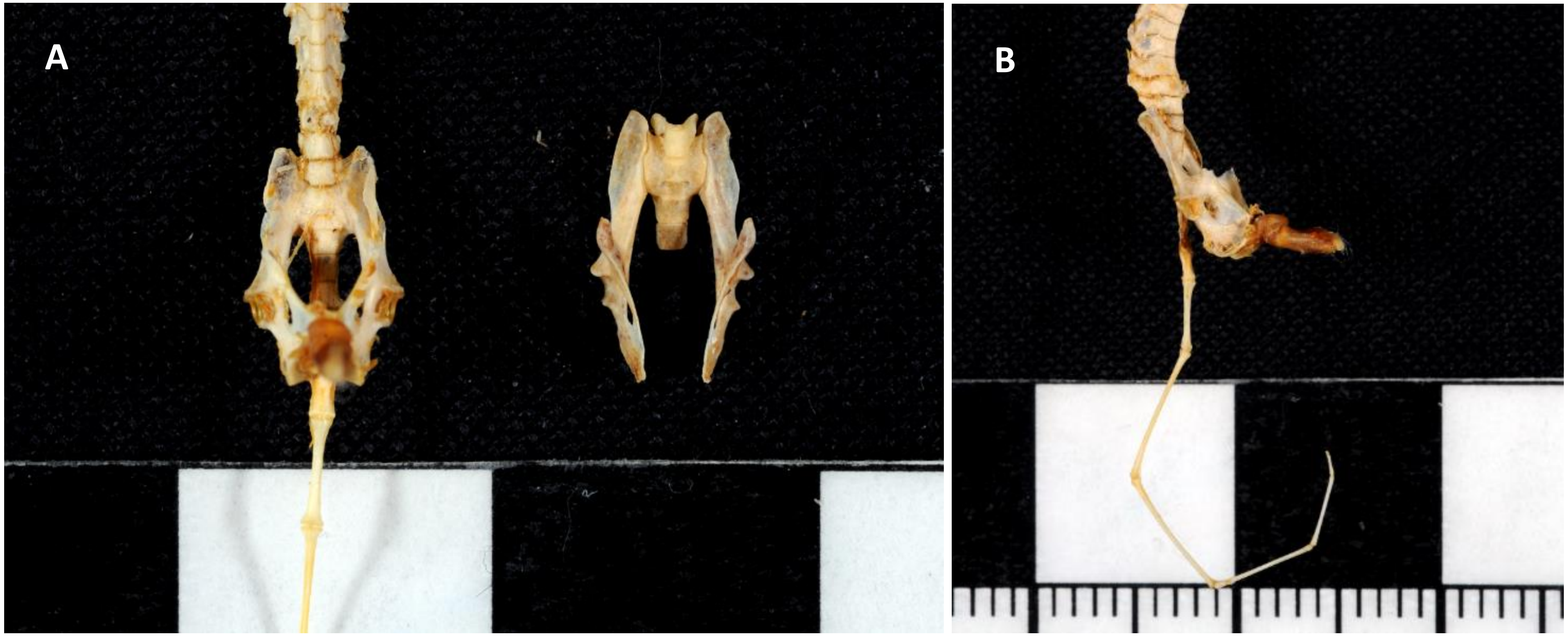

Figure S6. *Rhinolophus hipposideros* (Lesser horseshoe bat, Laurasiatheria: Chiroptera). B shows the male bat from A in lateral view to show the penis attached to the pelvis. NMW 29369 and NMW 52735.

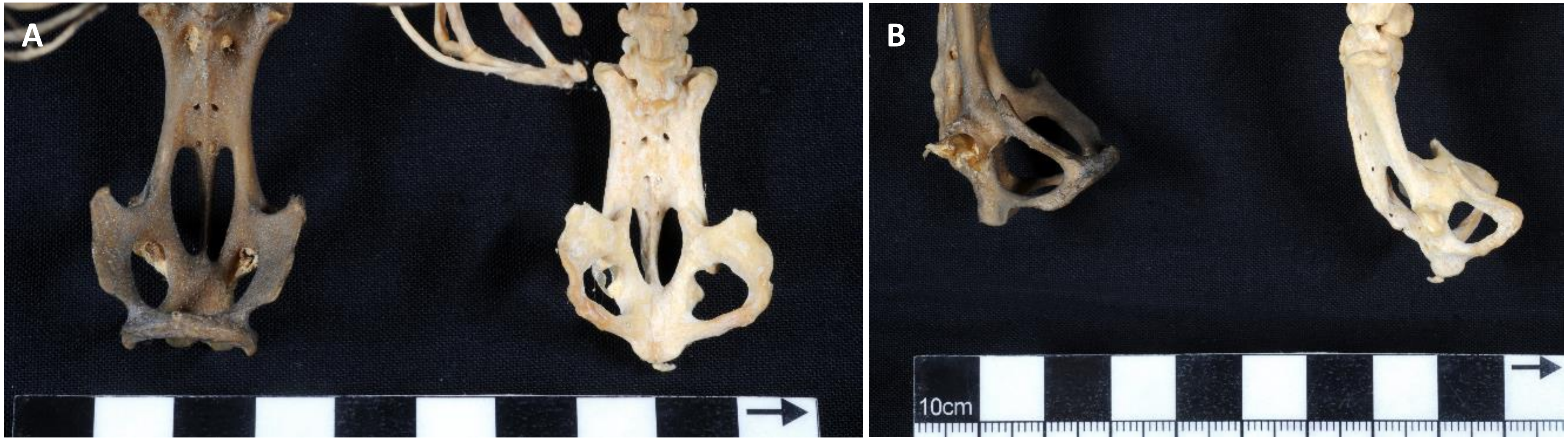

Figure S7. *Pteropus vampyrus* (Large flying fox, Laurasiatheria: Chiroptera). A: male and female pelves in ventral view; B: the same individuals in lateral view. NMW 957 and NMW 13524.

The wide-open pelvis is typical of female fruit bats.

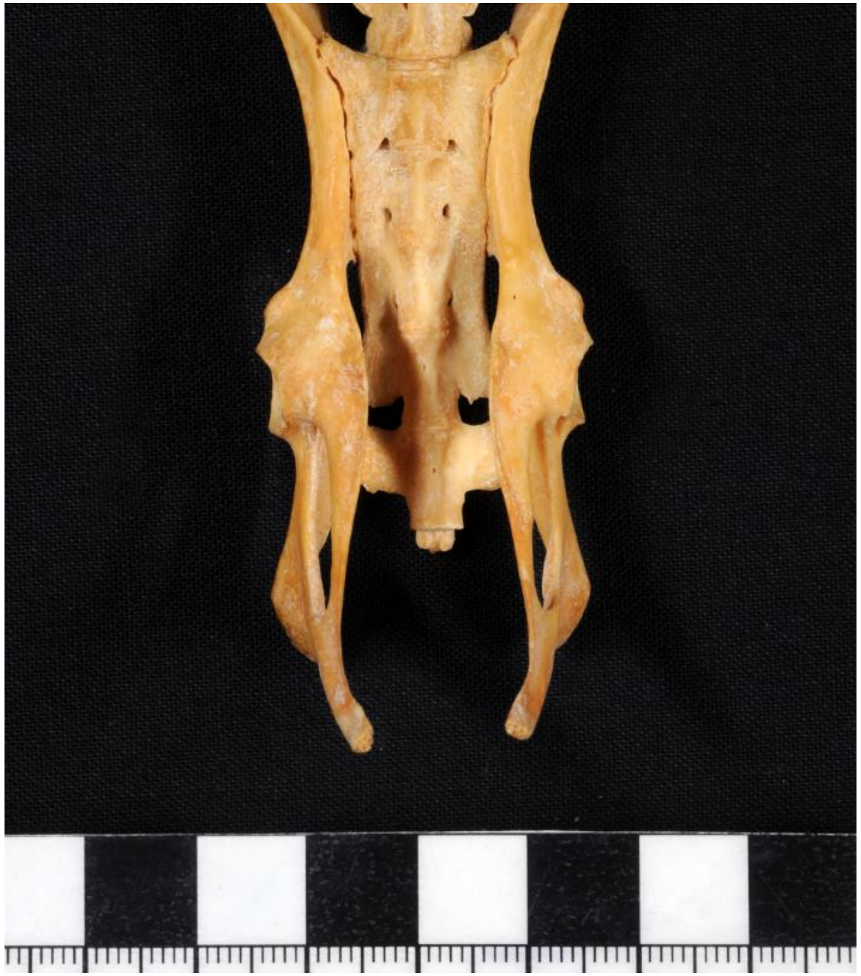

Figure S8. *Echinosorex gymnura* (Moonrat, Laurasiatheria: Eulipotyphla), female. NMW 21351. Hedgehog females show an open pelvis.

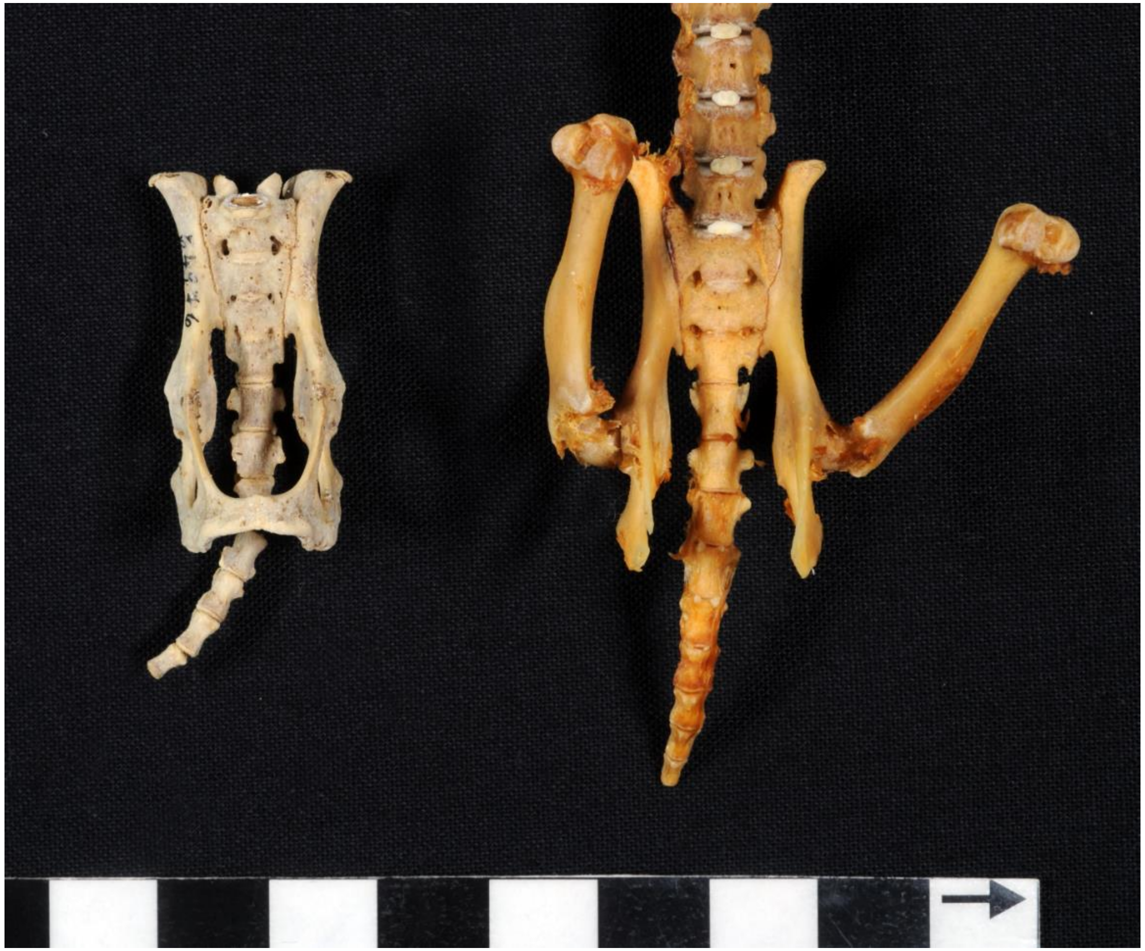

Figure S9. *Hemiechinus auritus* (Long-eared hedgehog, Laurasiatheria: Eulipotyphla). NMW 64976 and NMW 15185. The female pelvis is open.

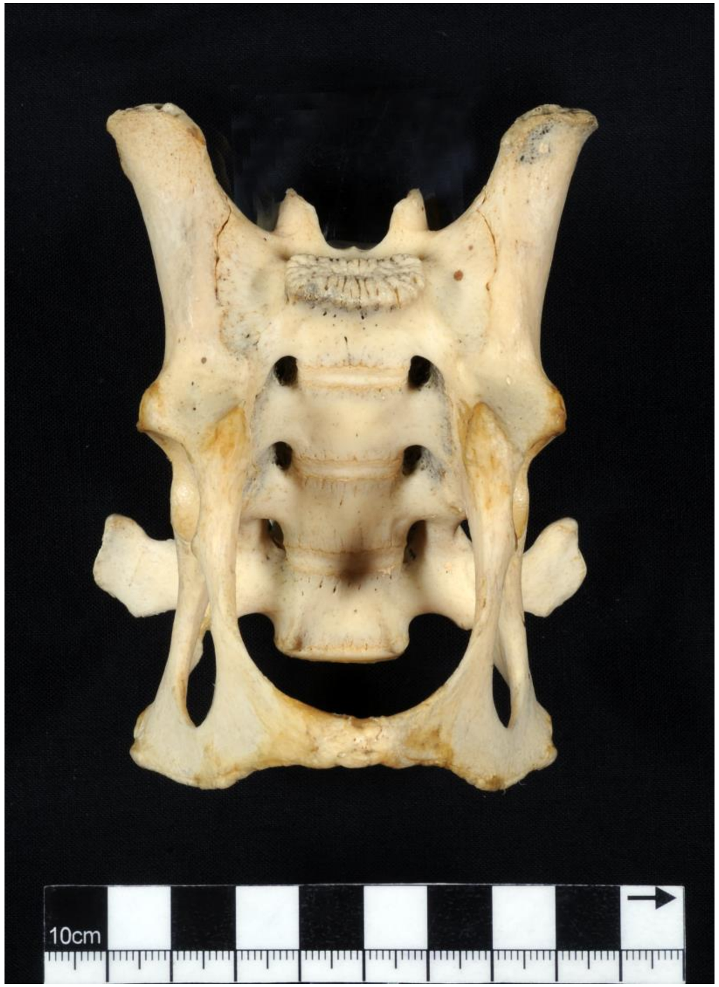

Figure S10. *Manis crassicaudata*, female (Indian pangolin, Laurasiatheria: Pholidota). NMW 2015.

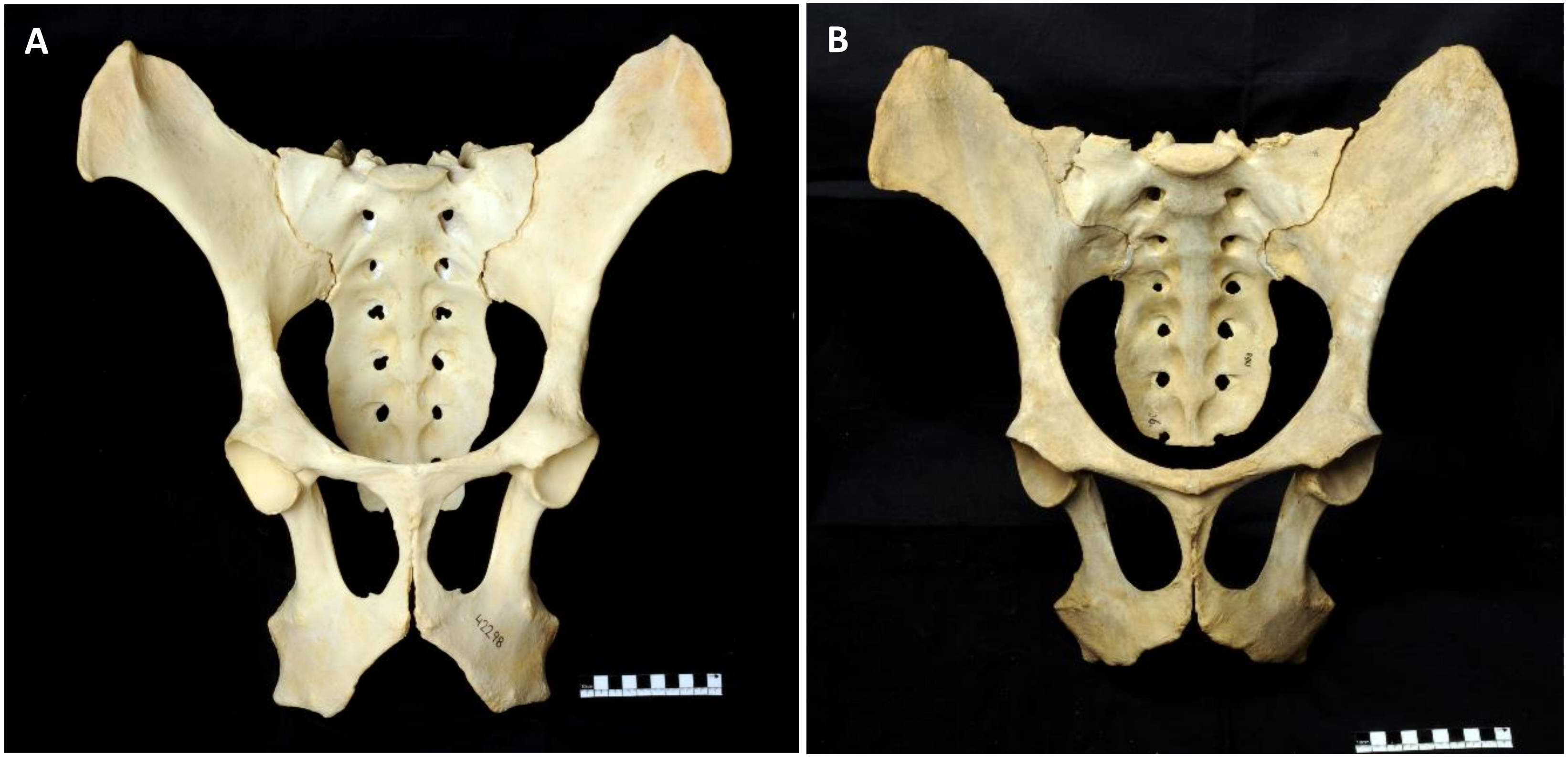

Figure S11. *Tapirus indicus* (Malayan tapir, Laurasiatheria: Perissodactyla). A: male (NMW 42298), B: female (NMW 1768).

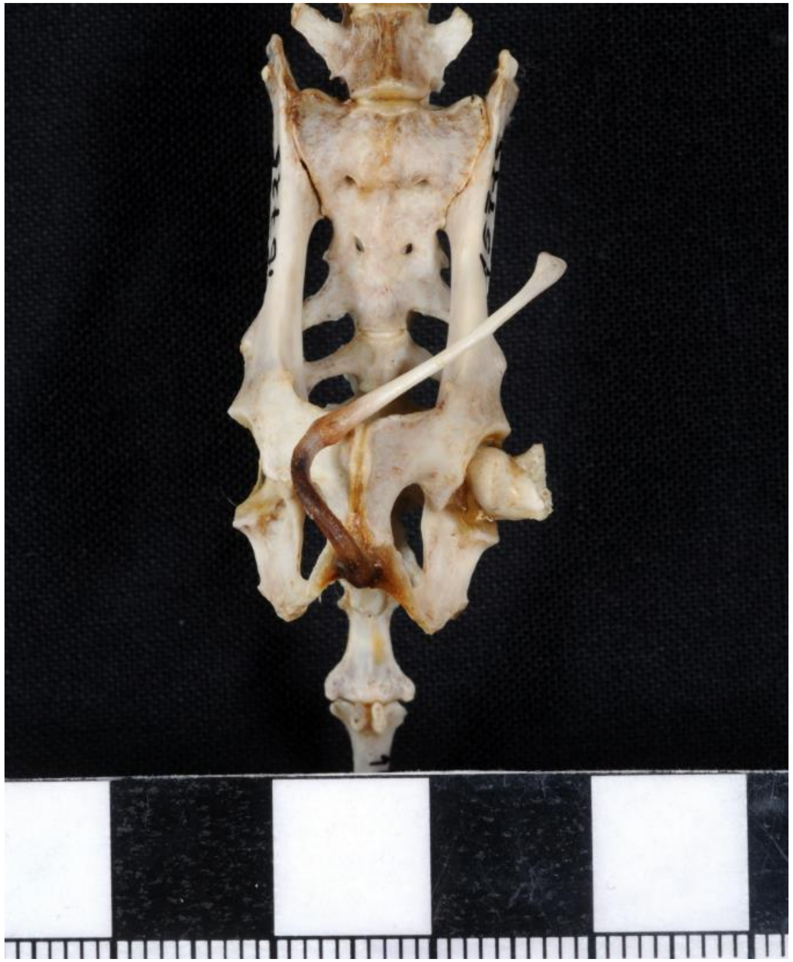

Figure S12. *Galago senegalensis*, male (Lesser bushbaby, Euarchontoglires: Primates). NMW 15725.
The penis is still attached to the pelvis. Note the long penis bone (baculum).

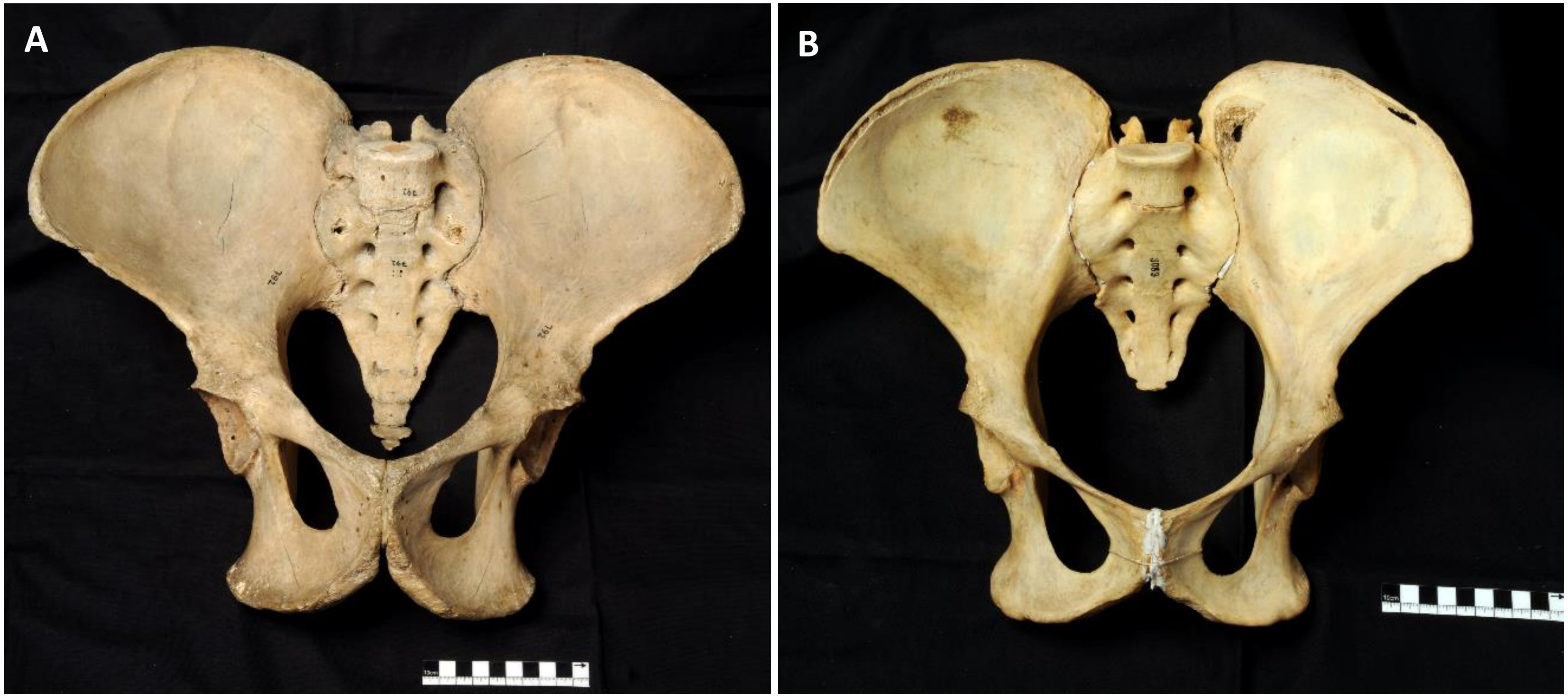

Figure S13. *Gorilla gorilla* (Gorilla, Euarchontoglires: Primates). A: male (NMW 792), B: female (NMW 3083).

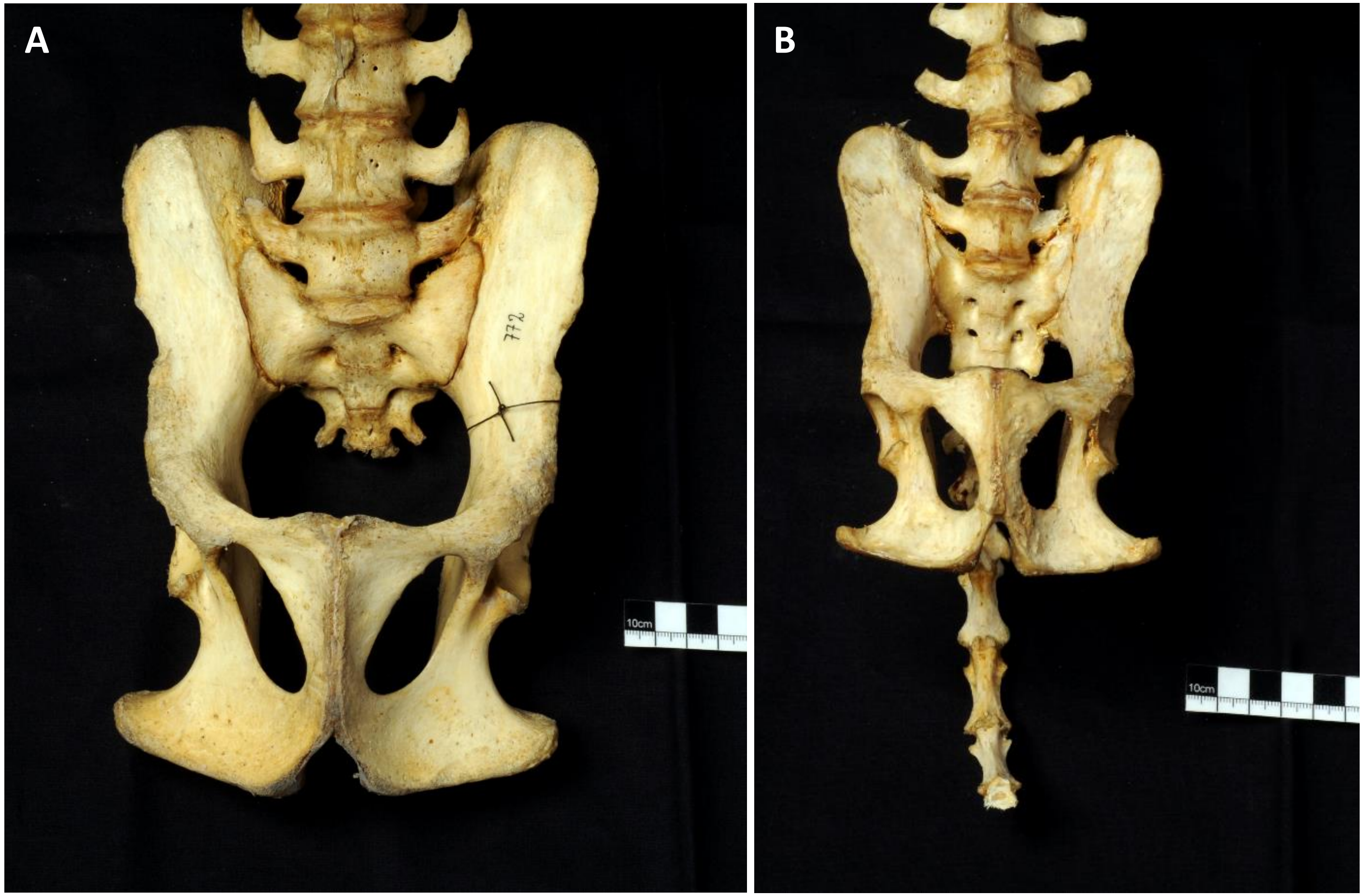

Figure S14. *Papio* sp. (Baboon, Euarchontoglires: Primates). A: male (NMW 772), B: female (NMW 825).

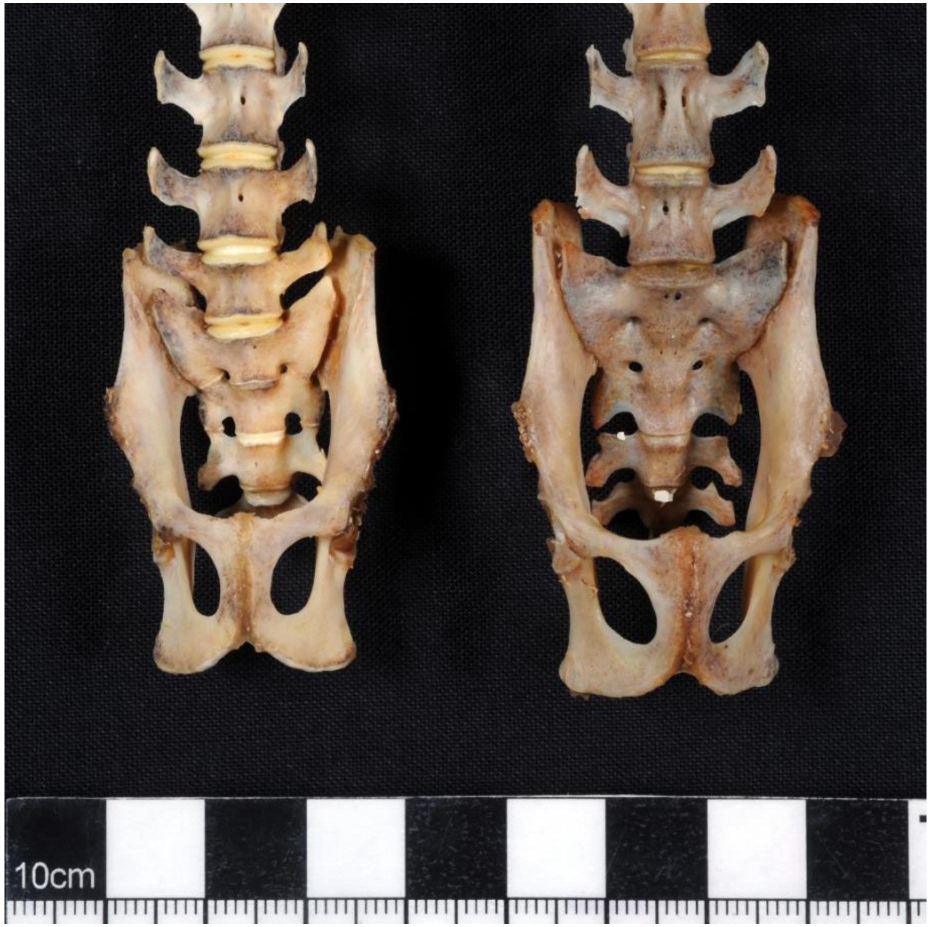

Figure S15. *Callimico goeldii* (Goeldi's marmoset, Euarchontoglires: Primates). NMW 62186 and NMW 62188. The male on the left is a subadult.

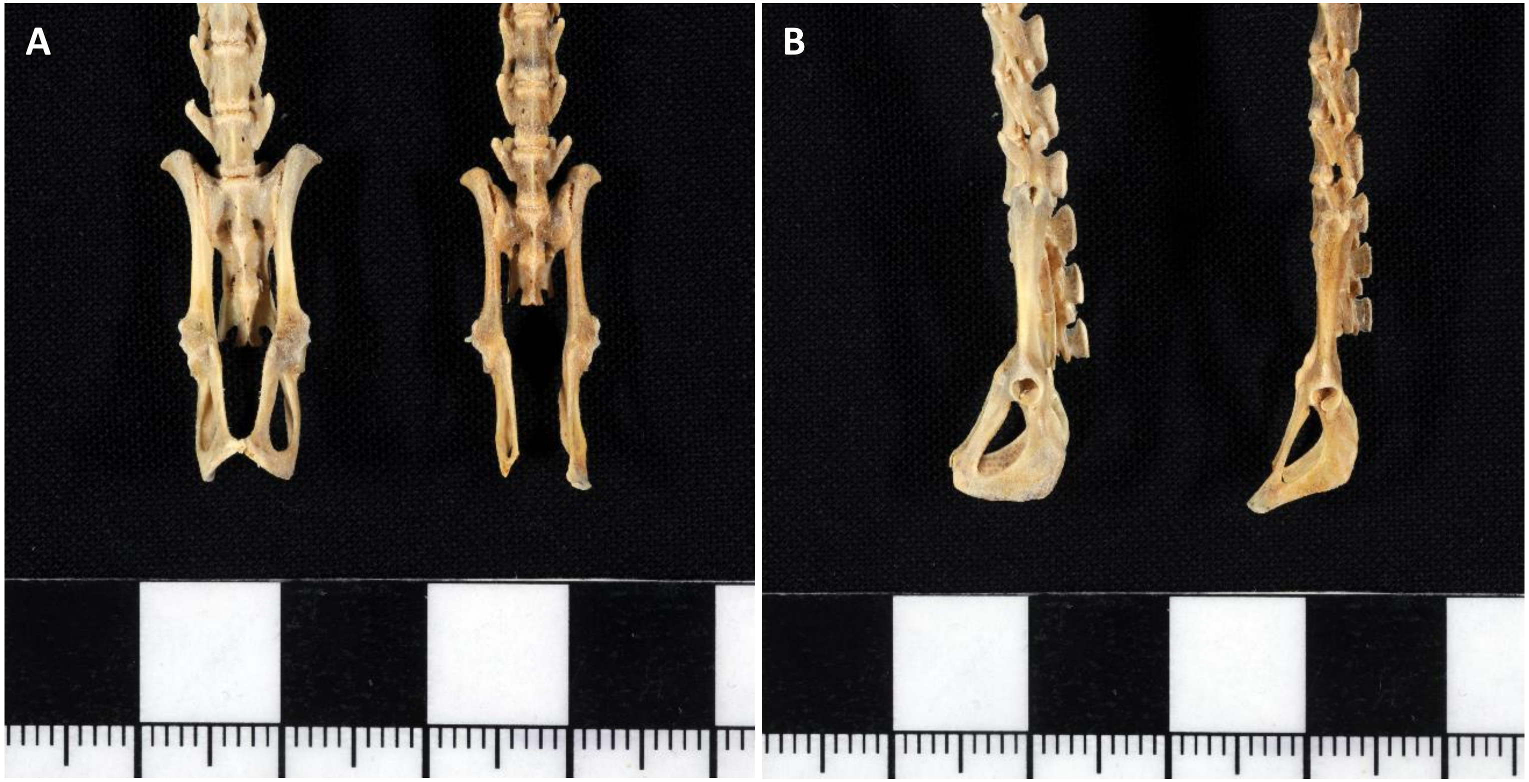

Figure S16. *Microtus oeconomus* (Tundra vole, Euarchontoglires: Rodentia). A: male and female pelves in ventral view, B: the same individuals in lateral view. NMW 9979 and NMW 9876.

This species of vole also has an open pelvis in the female sex. This is, however, not the rule in voles (personal observation).

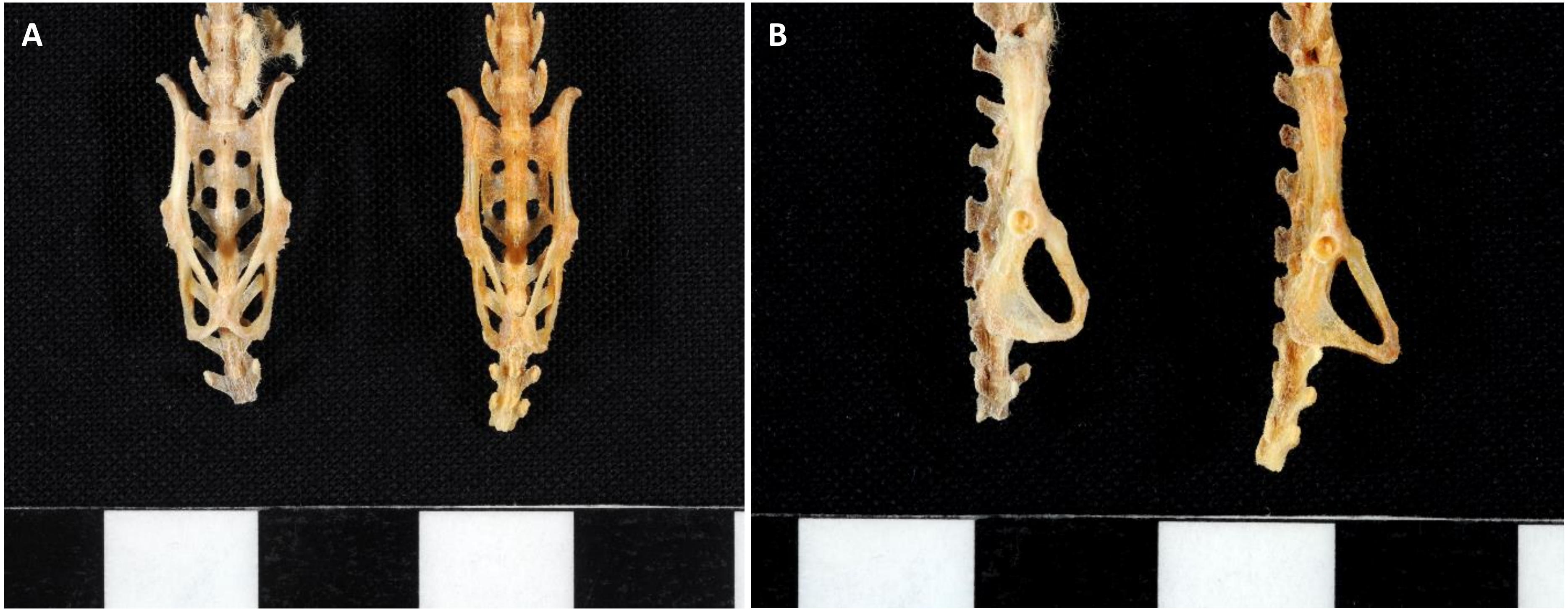

Figure S17. *Mus musculus* (House mouse, Euarchontoglires: Rodentia). A: male and female pelves in ventral view, B: the same individuals in lateral view. NMW 47783 and NMW 47784.

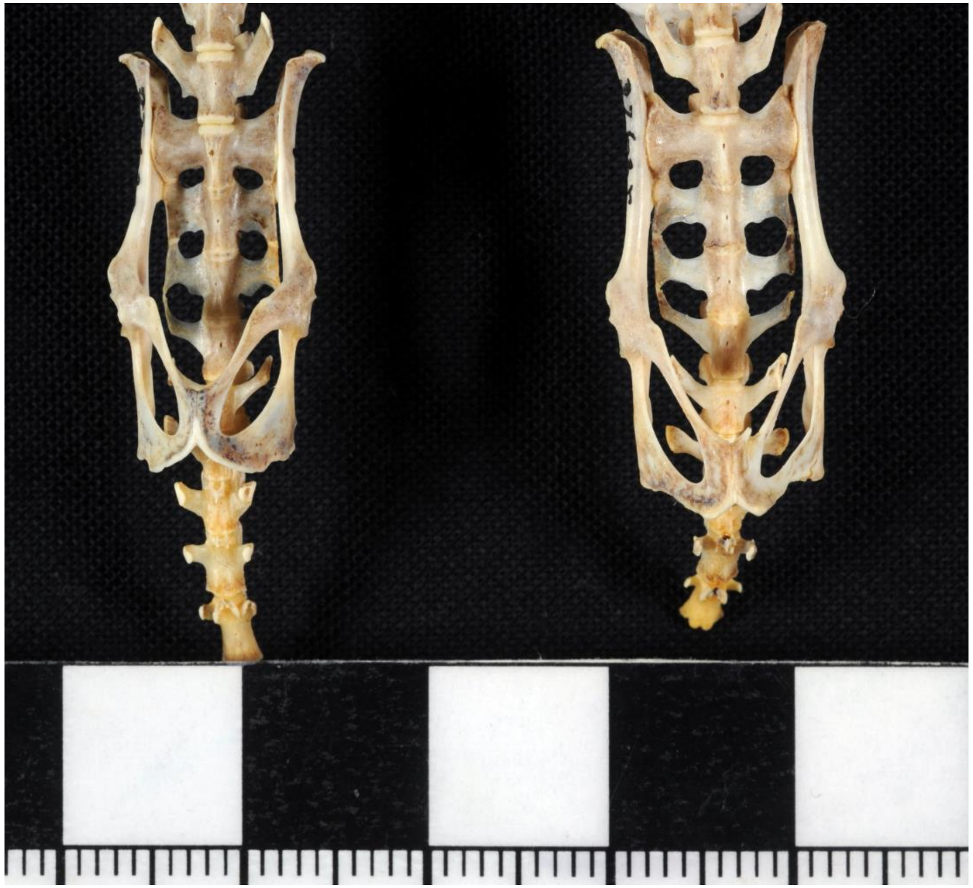

Figure S18. *Praomys* sp. (Soft-furred mouse, Euarchontoglires: Rodentia). NMW 32628 and NMW 32635.

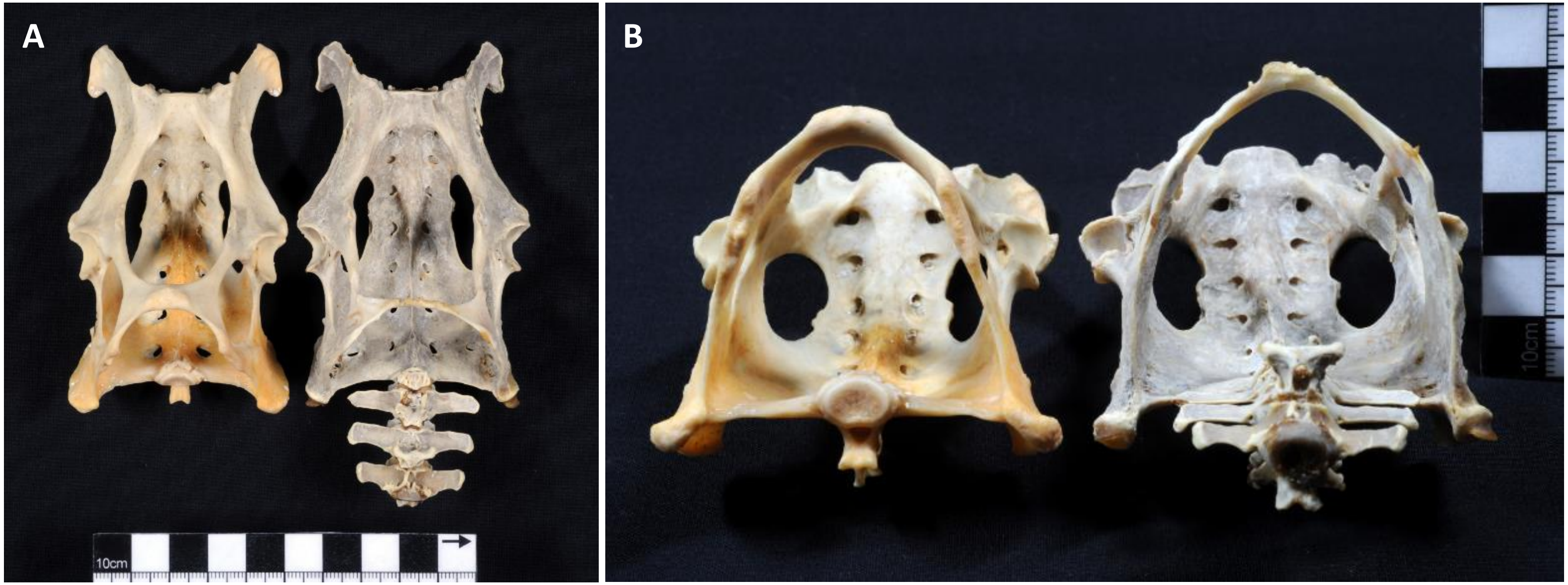

Figure S19. *Chaetophractus villosus* (Big hairy armadillo, Xenarthra: Cingulata). A: male and female pelves in ventral view, B: the same individuals in lateral view. NMW 38392 and NMW 15221.

Note the sexual dimorphism in the symphysis and the size of the pelvic canal.

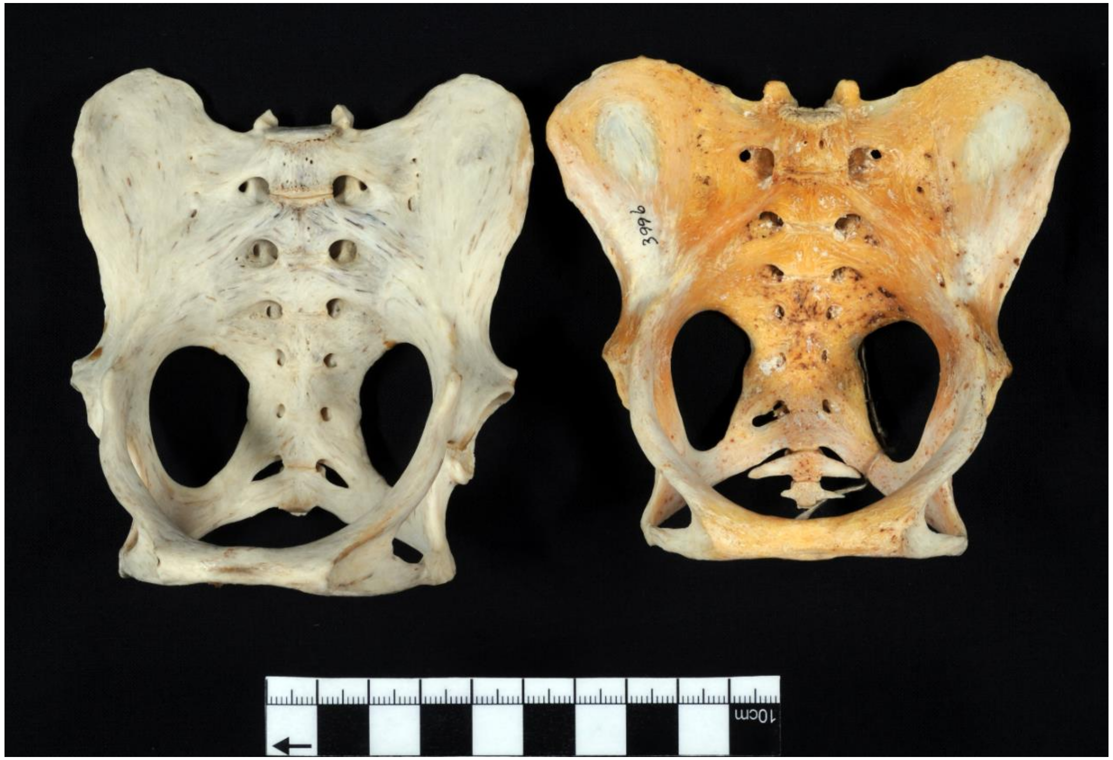

Figure S20. *Choloepus hoffmanni* (Hoffmann’s two-toed sloth, Xenarthra: Pilosa). NMW 31566 and NMW 3996.

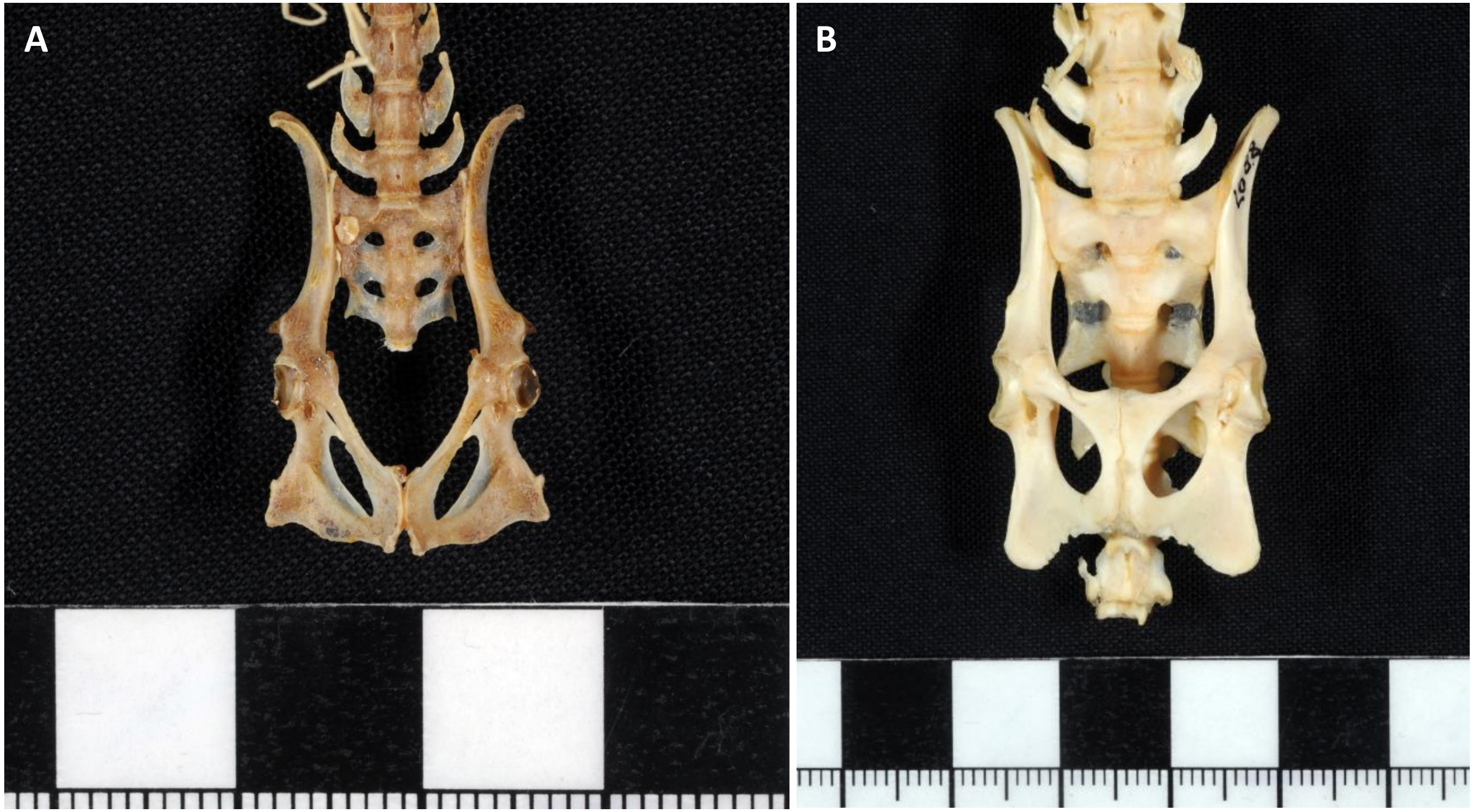

Figure S21. A: *Macroscelides proboscideus*, female (Round-eared elephant shrew) (NMW 57418), B: *Rhynchocyon stuhlmanni*, female (Checkered elephand shrew, Afrotheria: Macroscelidea) (NMW 8897).
