# Supplementary material for: Humans as inverted bats: A comparative approach to the obstetric conundrum
Source: Am J Hum Biol. 2019 Feb 27;31(2):e23227. doi: 10.1002/ajhb.23227 (PMC6492174; doi:10.1002/ajhb.23227)
Supplement: Supplementary file 3 — Table S2 Supporting information Table S2. The number of species by higher‐order taxon used to produce each Figure in the main text, including the data source. [file AJHB-31-na-s003.pdf]

Table S2

	Higher-order taxon	**Fig. 3**<br>Data from Boddy et al. 2012b<br>*species (N)*	**Fig. 4**<br>Data from Table S1<br>*species (N)*	**Fig. 5**<br>Data from Capellini et al. 2010b<br>*species (N)*	**Fig. 6**<br>Matching species b/w Boddy et al. & Cappelini et al.<br>*species (N)*
MONOTREMATA	Monotremata	3	0	0	0
PLACENTALIA	Afrosoricida	12	0	1	1
	Carnivora	60	52	15	9
	Cetartiodactyla	35	45	27	8
	Chiroptera	42	25	1	0
	Eulipotyphla	33	9	2	2
	Hyracoidea	1	1	1	1
	Lagomorpha	15	4	2	2
	Macroscelidea	3	0	2	0
	Perissodactyla	3	4	3	1
	Pholidota	0	2	0	0
	Primates	76	44	33	22
	Proboscidea	2	2	2	1
	Rodentia	258	93	18	10
	Scandentia	3	0	1	1
	Sirenia	1	1	0	0
	Xenarthra	9	2	1	1
MARSUPIALIA	Dasyuromorphia	18	0	0	0
	Didelphimorphia	13	0	0	0
	Diprotodontia	33	0	0	0
	Paucituberculata	1	0	0	0
	Peramelemorphia	9	0	0	0
	**Total**	**630**	**284**	**109**	**59**
